# Whole-exome sequencing reveals a novel homozygous mutation in the *COQ8B* gene associated with nephrotic syndrome

**DOI:** 10.1038/s41598-021-92023-3

**Published:** 2021-06-25

**Authors:** Mohd Fareed, Vikas Makkar, Ravi Angral, Mohammad Afzal, Gurdarshan Singh

**Affiliations:** 1 PK-PD Formulation and Toxicology Division, CSIR Indian Institute of Integrative Medicine, Canal Road, Jammu, 180001 India; 2 Academy of Scientific & Innovative Research (AcSIR), Ghaziabad, Uttar Pradesh 201002 India; 3 Department of Nephrology, Dayanand Medical College and Hospital, Ludhiana, Punjab 141001 India; 4 Visiting Consultant Renal Transplant, Dayanand Medical College and Hospital, Ludhiana, Punjab 141001 India; 5 Human Genetics & Toxicology Laboratory, Section of Genetics, Department of Zoology, Aligarh Muslim University, Aligarh, Uttar Pradesh 202002 India

**Keywords:** Consanguinity, Kidney, Kidney diseases

## Abstract

Nephrotic syndrome arising from monogenic mutations differs substantially from acquired ones in their clinical prognosis, progression, and disease management. Several pathogenic mutations in the *COQ8B* gene are known to cause nephrotic syndrome. Here, we used the whole-exome sequencing (WES) technology to decipher the genetic cause of nephrotic syndrome (CKD stage-V) in a large affected consanguineous family. Our study exposed a novel missense homozygous mutation NC_000019.9:g.41209497C > T; NM_024876.4:c.748G > A; NP_079152.3:p.(Asp250Asn) in the 9th exon of the *COQ8B* gene, co-segregated well with the disease phenotype. Our study provides the first insight into this homozygous condition, which has not been previously reported in 1000Genome, ClinVar, ExAC, and genomAD databases. In addition to the pathogenic *COQ8B* variant, the WES data also revealed some novel and recurrent mutations in the *GLA, NUP107, COQ2, COQ6, COQ7* and *COQ9* genes. The novel variants observed in this study have been submitted to the ClinVar database and are publicly available online with the accessions: SCV001451361.1, SCV001451725.1 and SCV001451724.1. Based on the patient's clinical history and genomic data with in silico validation, we conclude that pathogenic mutation in the *COQ8B* gene was causing kidney failure in an autosomal recessive manner. We recommend WES technology for genetic testing in such a consanguineous family to not only prevent the future generation, but early detection can help in disease management and therapeutic interventions.

## Introduction

Approximately 25% of CKD cases reveal a familial background in their inheritance^1,2^. Child and adolescent CKD cases mostly exhibit congenital anomalies of the kidneys and urinary tract (CAKUT), chronic glomerulonephritis, steroid-resistant nephrotic syndrome (SRNS), renal cystic ciliopathies, focal segmental glomerulosclerosis (FSGS) and nephrolithiasis or nephrocalcinosis. SRNS in children is one of the leading causes of progression to CKD stage-V or ESRD. Monogenic mutations account for about ~ 20% of individuals with early-onset CKD (before 25 years‑of‑age)^3^. CKDs with mendelian causes often differ substantially from acquired ones in their clinical prognosis, progression, and critical disease management, imposing a challenge in routine diagnostic processes. Moreover, CKD patients with stage-I to stage-III often remain asymptomatic and undetected until the patient reaches an advanced-stage disease (i.e., stage-IV to V).

Coenzyme Q8B (OMIM * 615567) glomerular nephropathy (*COQ8B*-GN) or nephrotic syndrome type 9 (NPHS9, OMIM # 615573) is an autosomal recessive type of CKD, inherited through allelic homogeneous variant or compound heterozygous variants. Recent studies have evidenced the monogenic mutations in *COQ8B*-GN predominantly manifested through SRNS, with proteinuria and typical FSGS renal histopathology^4–6^. The *COQ8B* is one of the several genes encoding enzymes required for the Coenzyme Q (ubiquinone; CoQ10) pathway. CoQ10, an essential cellular antioxidant, acts as an electron shuttle in the mitochondrial respiratory chain and a permeability transition pore regulator. Pathogenic mutations in any of the genes encoding enzymes of the CoQ10 biosynthetic pathway have been associated with nephrotic syndrome or CKD^7–10^.

Massive parallel sequencing (MPS) or next-generation sequencing (NGS) technologies help us conveniently to unravel the genetic basis of the disease, which could be used as a primary diagnostic tool for medical genetic testing. No detailed report of CKD at the genetic level available from Jammu & Kashmir, Northern India. Here, we employed the whole-exome sequencing (WES) approach to precisely identify the functional pathogenic mutations in a large consanguineous family affected with nephrotic syndrome (CKD stage-V or ESRD).

## Results

### Case presentation

**Figure 1:**
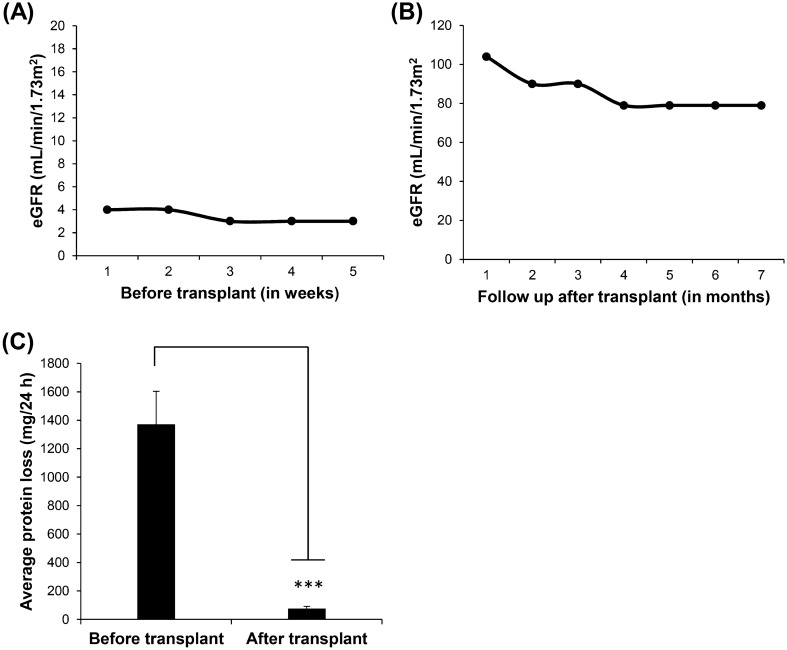
Pathophysiological characteristics of the proband (MC4, V-1). (**A**) Presenting the eGFR levels before dialysis treatment. (**B**) eGFR level after kidney transplant, showing the improvement in eGFR to the normal range. (**C**) Bar graph presenting the proteinuria (mean ± SE) before and after kidney transplant. The protein levels (mg/24 h) were considered only before the patient undergone dialysis. Mean (average protein loss) was calculated from three readings (intervals) in each category. ***p < 0.0001 statistically significant, using Student’s t-test.

This 25 years old lady (proband: MC4) was first detected to be having renal dysfunction during the third trimester of pregnancy with a serum creatinine of 13 mg/dL and hemoglobin level of 4 g/dL. Though the patient was hypertensive in the first trimester as well, she was not investigated further. On further evaluation, she was detected to be having normal size kidneys. However, it was decided not to go ahead with renal biopsy at that gestation and she continued on hemodialysis. She delivered a 1.5 kg baby and subsequently, her creatinine settled to around 3–4 mg/dL. She was lost to follow up and again presented with azotemic symptoms and serum creatinine of 21 mg/dL, five months down the line. The patient underwent live-related renal transplantation at the age of 23 years, with the donor being her father of age 45 years in 2018. Post-transplantation, the patient had an uneventful course except for an occasional episode of UTI. She has normal allograft function two years post-transplant with no complications and normal renal function. Her previous pregnancy was at the age of 21 years and she delivered at full term by cesarean section. She did not have a bad obstetric history. She underwent all the routine investigations during her first pregnancy and no abnormality was detected at that time. The detailed (follow up) report of estimated glomerular filtration rate (eGFR) and proteinuria in the MC4 (V-1) case has been displayed in Fig. 1.

The proband (MC4) had 05 siblings with 04 sisters and 01 brother. There is a family history of renal disease in two of her younger sisters (MC6 and MC7). Both of them were detected to have renal failure at the age of 15 and 16 years, respectively and subsequently became dialysis-dependent. Renal biopsy was not done in any of the siblings. Unfortunately, both of them succumbed to complications related to renal failure. The exact cause of death in both of them could not be ascertained.

### Identification of a pathogenic mutation using whole-exome sequencing

**Figure 2:**
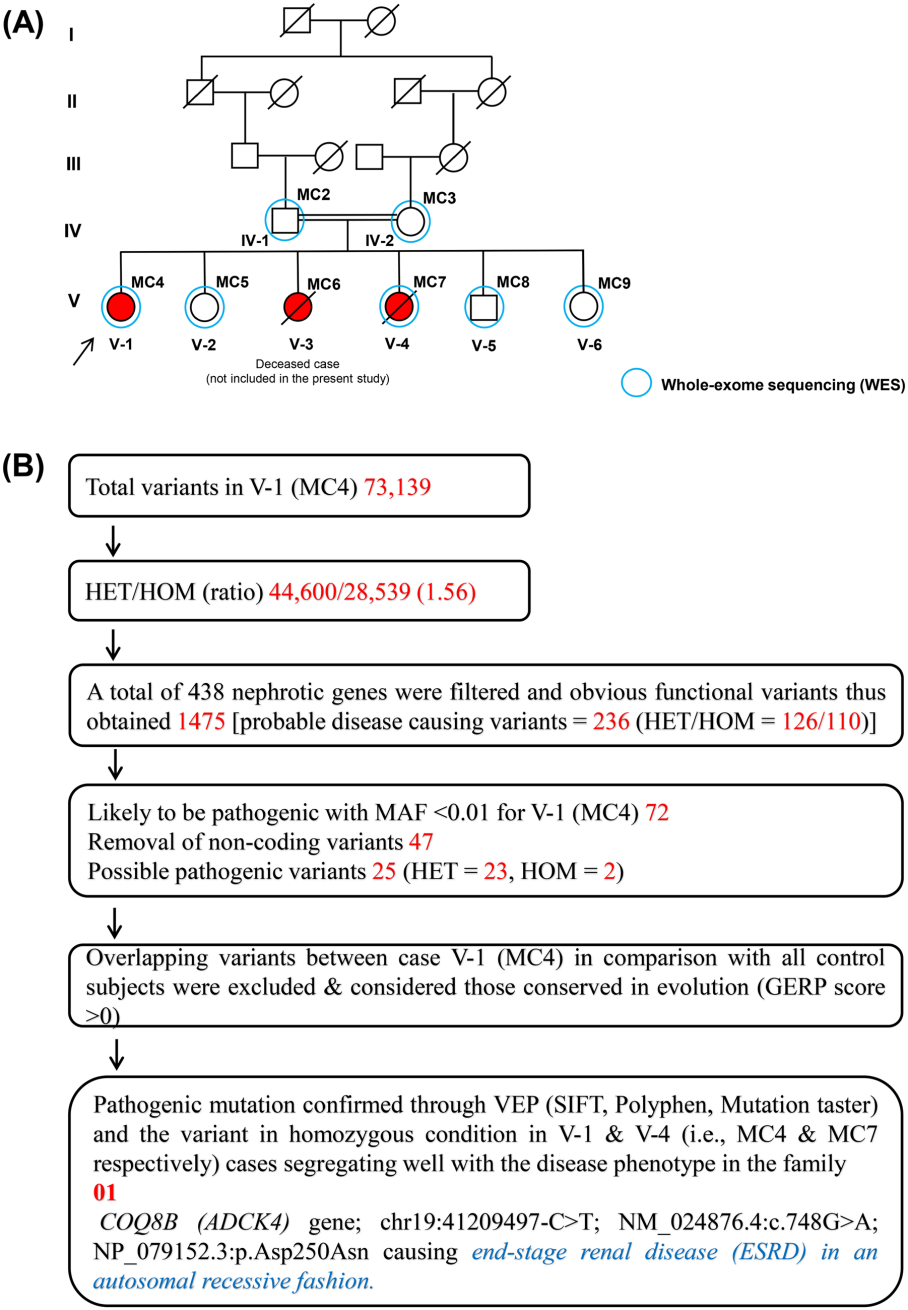
Family pedigree and Variant filtering strategy. (**A**) Family pedigree showing the inheritance pattern of nephrotic syndrome. Cases are shown in red color, and blue circles represent the subjects undergone WES (**B**) Variant filtering strategy. A narrowing down approach to identify the most promising causative mutation.

**Figure 3:**
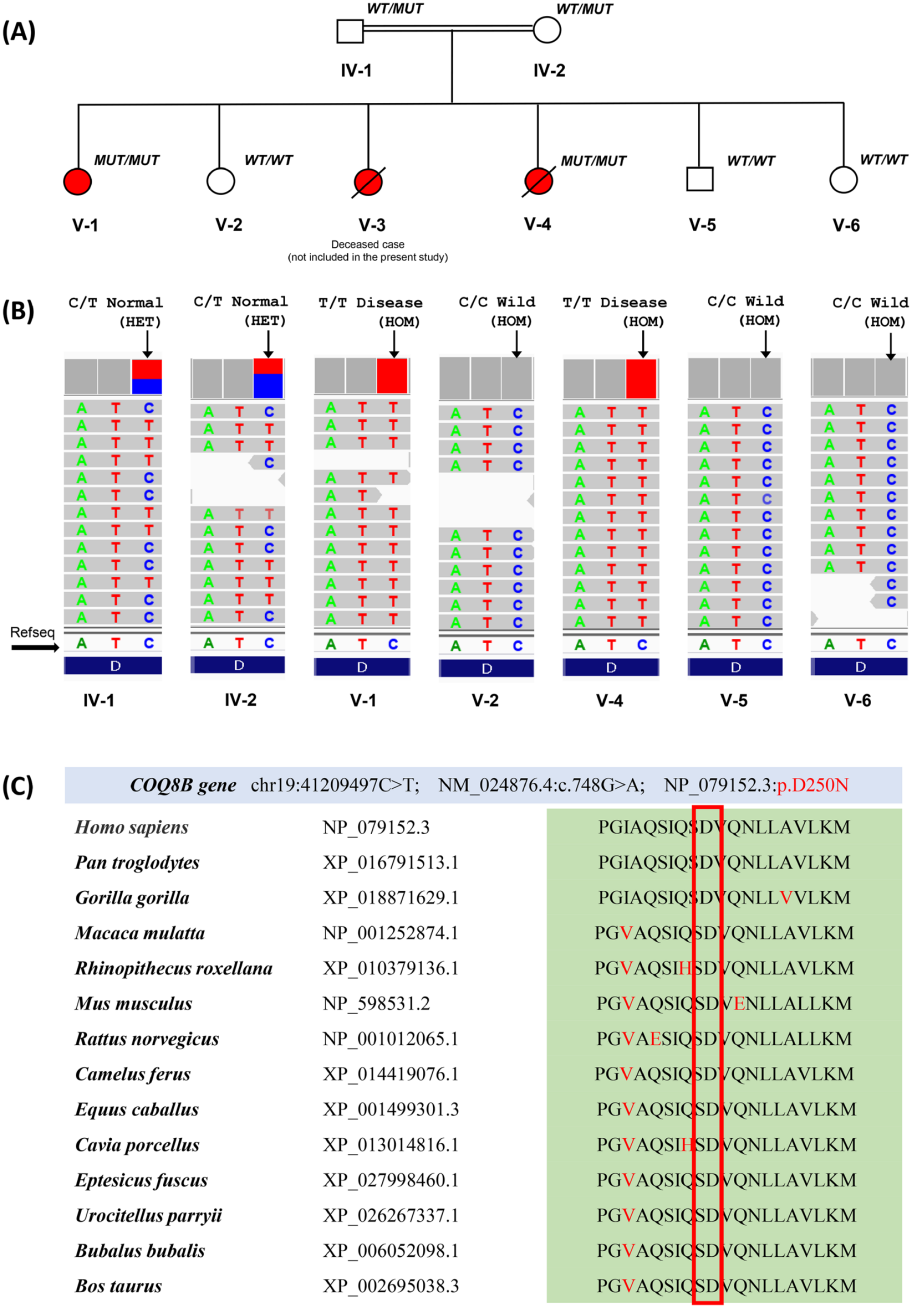
Candidate COQ8B variant co-segregation, variant validation using WES data and COQ8B protein conservation. (**A**) Pedigree showing the co-segregation of COQ8B variant (NM_024876.4:c.748G > A; NP_079152.3:p.D250N) in the family. Cases are presented in red color. (**B**) Whole-exome sequencing data (using IGV 2.5.3 software) reflects exactly the phenotypic data with homozygous cases (V-1 and V-4), heterozygous normal parents (IV-1 and IV-2) and three normal with homozygous reference allele (V-2, V-5 and V-6). (**C**) Comparing COQ8B protein for 14 vertebrates showed a highly conserved region at the mutation site (Asp250Asn).

**Figure 4:**
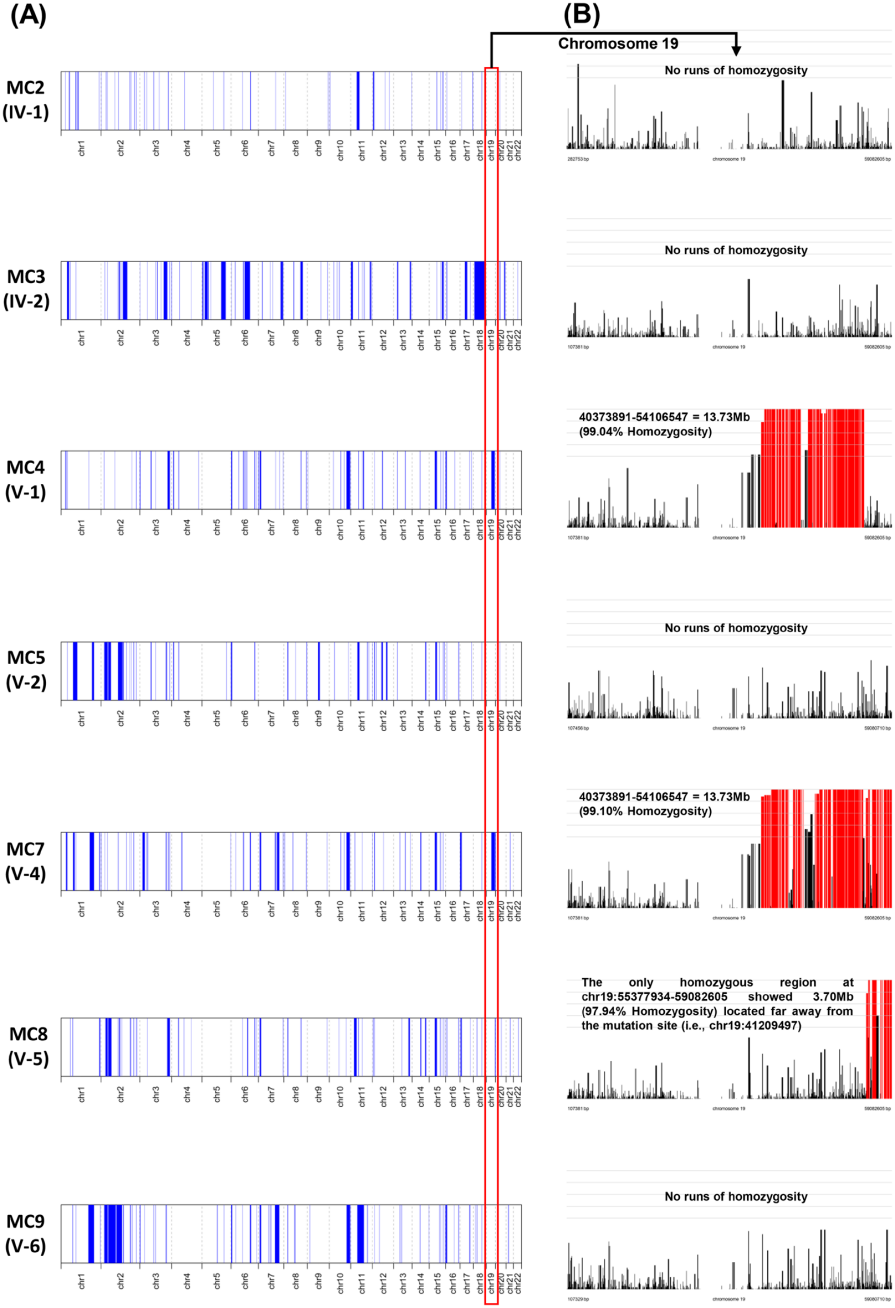
Runs of homozygosity (ROH). (**A**) Showing the distribution of homozygous regions (blue blocks) in the autosomes. (**B**) Exposition of ROH on chromosome 19. The histograms highlighted in red color represent the estimated ROH.

The whole-exome sequencing produced approximately 73,000 overall pass-on-target variants for all seven samples (Supplementary Table S1). Based on the filtering strategy, the variant statistics and a summary of probable disease-causing have been presented in Supplementary Tables S2–S5. Figure 2B illustrates the flowchart of the variant filtering strategy used to find out the most promising causative mutation. Using online GenIO (based on RefGene, NHLBI-ESP, 1000Genomes, dbSNP, ClinVar, COSMIC, gnomAD, OMIM and M-CAP databases)^11^ and mutation distiller (based on HPO, OMIM, OrphaNet and ACMG actionable genes databases)^12^ integrated pipelines, we observed 72 likely pathogenic variants with MAF < 0.01 (Supplementary Table S5). Upon exclusion of non-coding and overlapping variants with control subjects and considered those conserved in evolution (GERP score > 0), the pathogenic variant (*COQ8B* gene, chr19:41209497:C > T; NM_024876.4:c.748G > A; NP_079152.3:p.Asp250Asn) with reference genome GRCh37/hg19 was observed in the homozygous condition and co-segregated with the disease phenotype in the family (Fig. 3A). The mutation was confirmed using BAM and VCF files in IGV 2.5.3 software^13^ (Fig. 3B and Supplementary Fig. S1). Figure 4 shows the distribution of runs of homozygosity (ROH) among all individuals of the family. Upon delineating the region of interest (chr19:41209497:C > T) on chromosome 19, we found the two cases (MC4 and MC7) exhibit the ROH > 13 Mb at position chr19:40373891-54106547, while the rest of the individuals do not present ROH regions covering the candidate mutation. The ROH data provides evident detail for disease-causing mutation harboring in the homozygous region of the two children of a consanguineous union, inherited through an autosomal recessive manner.

The in silico evaluation of the candidate variant (c.748G > A/p.D250N) using VEP tools (CADD, FATHMM, MetaLR, MetaSVM, MutationTaster, PolyPhen-2 and SIFT) confirmed its deleterious impact and predicted to be disease-causing in all of its coding transcripts (ENST00000324464.3, ENST00000243583.6, ENST00000450541.1, ENST00000595254.1). Moreover, the observed rare variant (NM_024876.4:c.748G > A, NP_079152.3:p.D250N, rs769834604) with the homozygous condition has no previous records in the genomAD, ExAC, 1000Genome and ClinVar databases. Our study provides the first report of *COQ8B*:c.748G > A (p.D250N) pathogenicity in the ClinVar database (accession: SCV001451361.1).

In addition to the pathogenic *COQ8B* variant, we surprisingly found some novel variants in *GLA* (NM_000169.2:c.473C > T; p.T158I) and *NUP107* (NM_020401.2:c.1781G > A; p.C594Y) genes and have been submitted to ClinVar database with accessions SCV001451725.1 and SCV001451724.1 respectively. Our study has also revealed some known missense mutations in the *COQ2, COQ6, COQ7* and *COQ9* genes involved in the CoQ10 biosynthesis pathway.

## Discussion

Our study exposed the adolescence onset *COQ8B* nephropathy with clinical features characterized by absence of hematuria, moderate to severe proteinuria, edema and chronic renal failure making it unique to the congenital glomerulopathies. The studied patients remained asymptomatic at an early age and peculiar symptoms of the advanced CKD appeared at the time of diagnosis. The genetic study of the present consanguineous family revealed the heterozygous mutation in *COQ8B* among parents leading to the homozygous offspring in an autosomal recessive inheritance. The homozygous mutation (p.D250N) in cases co-segregated well with the nephrotic phenotype in the family. Several other studies have also reported different genetic substitutions at locus chr19:41209497 of the *COQ8B* gene causing nephrotic syndrome (Table 1).

**Table 1:** Genotypic and phenotypic characteristics of *COQ8B* variants (at position chr19:41209497) in children.

Family (cases)	*c*DNA change	*AA* change	Zygosity	Phenotype	Renal histopathology	Treatment	Ethnic group	References
7 (2)	c.748G > A	p.Asp250Asn	Homozygous	SRNS	ND	Transplant in 01 case	Indian	Current study
2 (1)	c.748G > C	p.Asp250His	Heterozygous	SRNS	FSGS	ND	Chinese	^14^
3 (1)	c.748G > C	p.Asp250His	Heterozygous	SRNS	Sclerosing glomerulonephritis	ND	Chinese	^14^
6 (1)	c.748G > C	p.Asp250His	Homozygous	SRNS	NS	ND	Chinese	^14^
7 (1)	c.748G > C	p.Asp250His	Homozygous	SRNS	FSGS	ND	Chinese	^14^
8 (1)	c.748G > C	p.Asp250His	Homozygous	Proteinuria	FSGS	ND	Chinese	^14^
1 (1)	c.748G > C	p.Asp250His	Heterozygous	Proteinuria	ND	CoQ10	Chinese	^5^
1(1)	c.748G > C	p.Asp250His	Heterozygous	Proteinuria	MGA	ND	Chinese	^15^
1(1)	c.748G > T	p.Asp250Tyr	Heterozygous	Proteinuria	FSGS	CoQ10	Belgian	^16^

*ND* Not done or no data, *NS* nephrotic syndrome, *FSGS* focal segmental glomerulosclerosis, *MGA* minor glomerular abnormalities.

Most of these studies have observed c.748G > C (p.D250H) and a few c.748G > T (p.D250Y)variations linked with SRNS and proteinuria with clinical FSGS/ or glomerular abnormalities. Our study provides the first insight into the homozygous condition of c.748G > A (p.D250N) with similar pathophysiological observations.

The *COQ8B* (OMIM * 615567), previously termed as *ADCK4* (encoding the *aarF domain‐containing protein kinase 4*), belongs to the protein kinase superfamily containing a conserved domain with the catalytic activity of protein kinases. The co-enzyme Q8B encoded by the *COQ8B* gene (chromosomal position: 19q13.2a) is a mitochondrial matrix protein tangentially associated with the inner membrane. The protein contains 544aa in humans and is characterized by a single-pass transmembrane with a molecular weight of 60,069 Da. The *COQ8B* encoded protein plays a regulatory function in the biosynthesis of CoQ10. There have been 29 mutations reported in Human Gene Mutation Database (HGMD), including missense, nonsense and indels, mostly showing the autosomal recessive inheritance pattern for nephrotic disorders. Reports of several mutations in the *COQ8B* gene have been involved in damaging the kidney function^4,7,17–19^. The expression of *COQ8B* in the mitochondria of podocytes, renal tubules and collecting ducts is clear with a well-established corresponding link toward renal homeostasis hallmarks. A recent study provides the genetic and pathophysiologic evidence for the *COQ8B* in podocytes function. The in vitro experiments revealed a significant reduction of CoQ10 concentration in the *COQ8B*-knockout podocytes, leading to decreased respiratory chain activity, mitochondrial potential and consequently showing increased dysmorphic mitochondria^20^.

Our study revealed the novel and rare pathogenic mutation (NM_024876.4:c.748G > A (p.Asp250Asn) in the highly conserved region of exon 9 of the *COQ8B* gene (Fig. 3C and Supplementary Fig. S2), which is one of the 16 different regulatory genes (*PDSS1, PDSS2, COQ2, COQ3, COQ4, COQ5, COQ6, COQ7, COQ8A, COQ8B, COQ9, COQ10A, COQ10B, FDX1L, FDXR, and ALDH3A1*) ubiquitously required for the CoQ10 biosynthesis pathway in mammals^4,8,9,21^. The pathogenic mutations, including missense/non-sense, indels and frameshifts, could lead to loss of COQ8B kinase function, ultimately affecting the COQ10 biosynthesis pathway^22^.

**Figure 5:**
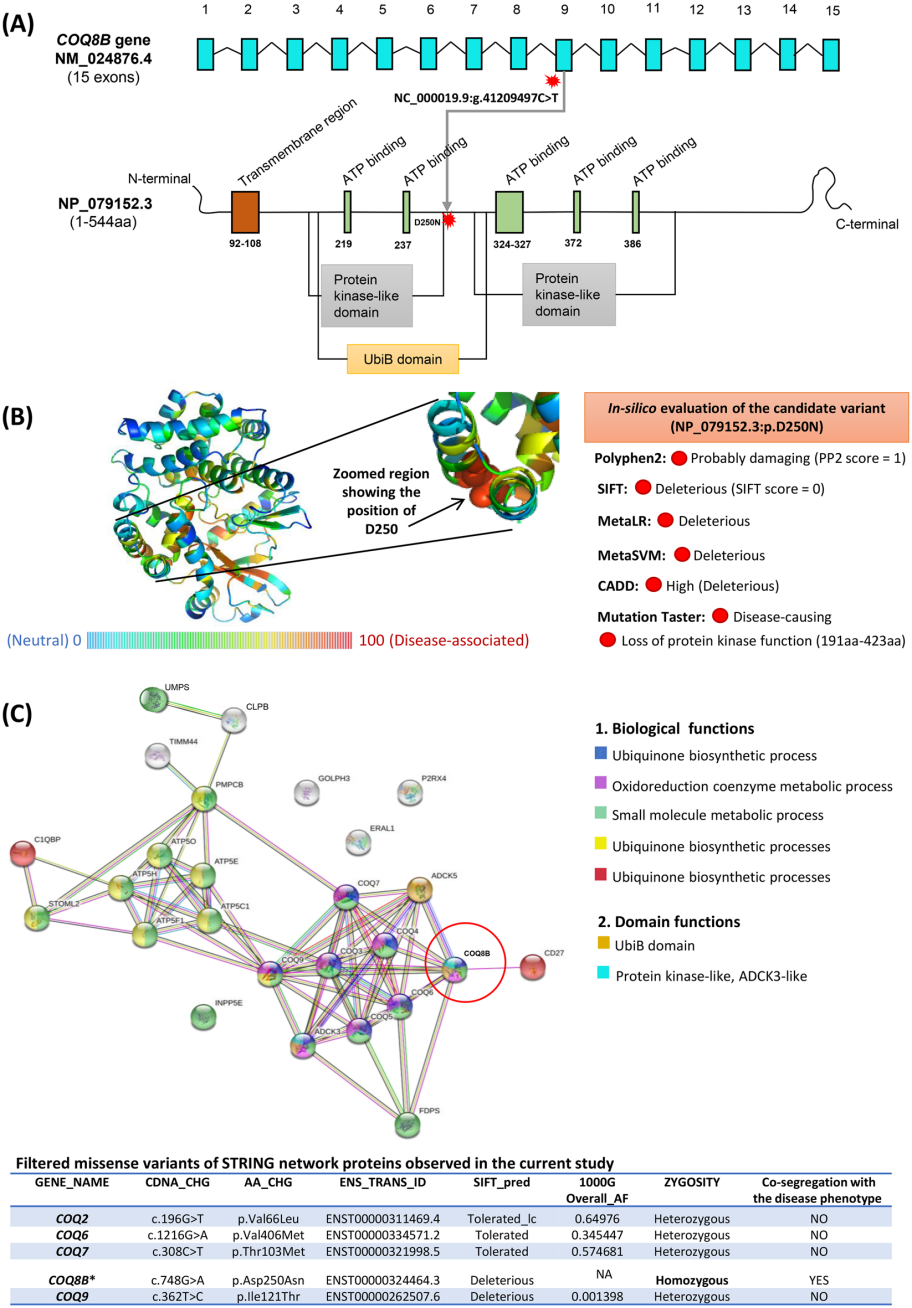
*COQ8B* gene and its transcript, three-dimensional structure of COQ8B and STRING network. (**A**) Showing the details of the *COQ8B* gene (15 exons) and the expressed protein (using neXtProt, online database)^24^. The pathogenic mutation (NC_000019.9:g.41209497C > T) in the 9th exon leading to the replacement of Aspartic acid (negatively charged) by Asparagine (non-charged) amino acid at position 250. (**B**) Structural model of COQ8B with the exposition of candidate variant position (D250), using SuSPect prediction algorithm^25^. Multiple in silico algorithm (shown on the right) data reflect the candidate mutation to be deleterious and disease-causing. (**C**) Network showing the functional association of COQ8B protein using STRING v11.0 (https://stringdb.org/). The proximity of the proteins via threads predicts the association and their role in the COQ10 biosynthesis pathway. In addition to the candidate pathogenic variant in the *COQ8B* gene, we have also identified the variants in *COQ2, COQ6, COQ7* and *COQ9* genes for the current STRING model.

Figure 5A,B provided the comprehensive report of the *COQ8B* gene with its expressed transcript (containing functional domains) and predicted the 3-dimensional structure of the protein with the precise exposition of the candidate variant impact. The protein–protein interaction with biological processes and domain functions (using STRING v11.0)^23^ of COQ8B has been displayed in Fig. 5C. A recent study unraveled the COQ8B interactions with mitochondrial proteins such as COQ5, as well as cytoplasmic proteins such as myosin and heat shock proteins. They observed a decreased COQ5 level in the *COQ8B*-knockout podocytes, but overexpression of *COQ8B* transfected with wild-type COQ8B rescued the COQ5 level^20^. We assume the candidate pathogenic mutation (*COQ8B:*p.Asp250Asn) identified in the present study disrupts the protein–protein interaction via COQ5 in the CoQ megacomplex, leading to progressive renal damage and consequently ESRD. In addition to the candidate variant, our study also revealed multiple known mutations in the STRING model (*COQ2*:p.Val66Leu, *COQ6*:p.Val406Met, *COQ7*:p.Thr103Met and *COQ9*:p.Ile121Thr) involved in the CoQ10 biosynthesis pathway (Fig. 5C).

Multiple in silico tools confirmed the deleterious and possibly damaging impact of the candidate variant [NM_024876.4(*COQ8B*):c.748G > A (p.Asp250Asn)]. The observed candidate variant harbor the most conserved region of the *COQ8B* gene across different vertebrates (Supplementary Fig. S2a). Using NCBI database, the expression profiling of the *COQ8B* gene of 16 human tissue samples provided substantial evidence for its active role in kidney function (Supplementary Fig. S2b). Multiple in silico programs confirmed the pathogenicity candidate variant. However, functional studies using in-vivo models will be carried out in the future.

In podocytes, the expressed *COQ8B* interacts with other members of the COQ10 biosynthesis pathway and the mutations in the gene have been linked with steroid-resistant nephrotic syndrome (SRNS)^4^. The biological functions and domains feature depicts the importance of COQ8B as one of the significant components of the COQ10 biosynthesis pathway. The kinase activity of COQ8B allows it to interact selectively and non-covalently with ATP, involved in various metabolic processes^22^. The COQ10 (coenzyme Q10) a lipid-soluble electron carrier, specifically involved in the electron transport chain that exerts antioxidant, anti-inflammatory and present favorable cardiac, hypertension, lipid and glucose metabolic functions^26^. In the progression of CKD, the oxidative stress, inflammation and endothelial dysfunction biomarkers are easily detectable^27^. COQ10 supplementation has also been used to restrain oxidative stress in CKD patients^28^. In the present study, the delayed genetic diagnosis hindered the possible use of CoQ10 supplementation to the patients.

The genotypic data of *COQ8B* among offspring present all the homozygous genotypes. Interestingly, there were no heterozygous children, although the chance of this was supposed to be twice of a homozygous state in each pregnancy. The lack of heterozygous genotypes in the offspring and the presence of only homozygous genotypes (*WT/WT* or *MUT/MUT*) among the offspring seems most likely due to chance in this family.

## Conclusion

Our study provides the first report of *COQ8B* homozygous variant NM_024876.4:c.748G > A (p.Asp250Asn) in a family affected with idiopathic end-stage kidney disease. The variant p.Asp250Asn was co-segregated with the disease phenotype and the in silico evaluations confirmed its pathogenicity. Based on the clinical and genetic findings, we assume SRNS (NPHS9) would have led these patients to the most advanced CKD (stage-V). Due to consanguineous marriage between parents, the autosomal recessive effect of *COQ8B* appeared in their generation. Our study provides a piece of safeguard advice for clinicians to make people familiar with the genetic cause and its increased risk in the context of consanguineous marriages. The familial and premarital screening will help in controlling the *COQ8B*-related nephrotic syndrome in future generations. Identification of such mutations supports better management of such disorders through genetic counseling and appropriate diagnosis could open new gateways for precision medicine. Early detection of *COQ8B* pathogenic mutations in such cases could better help disease management by adopting personalized therapy using CoQ10 supplements.

## Materials and methods

### Ethics statement

The study was approved by Institutional Ethics Committee (IEC), Dayanand Medical College and Hospital (DMCH), Ludhiana, India. The informed written consent was also obtained from the subjects involved in this study. On behalf of children under the age of 18 years, we obtained the informed written consent from the parents. The study was performed following the guidelines and regulations of the Indian Council of Medical Research (ICMR), New Delhi.

### Study design

Earlier studies have evidenced a high prevalence of consanguinity among Muslim populations of Jammu and Kashmir, North India^29–33^. A large consanguineous Muslim family was identified from the Poonch District of Jammu and Kashmir. The family comprised of 08 members and a detailed pedigree up to three generations back provided the evidence for no prior history of kidney disease (Fig. 2A).

Out of 06 children, 03 females were diagnosed with advanced CKD/ ESRD (stage-V) at 16–23 years. Two females died within a year on dialysis treatment. One eldest female survived after a kidney transplant from her father. The medical records of all family members were examined. We collected blood samples from 7 members (02 cases and 05 controls) only, while one female (MC6) died before we could plan this study. The detailed history and disease manifestation based on medical records has been presented in Supplementary Fig. S3.

### Sample collection, DNA isolation and quantification

The blood samples were collected and DNA was extracted using QIAamp DNA Mini Kit 50 (Cat# 51304, Qiagen). The DNA samples were was subjected to QIAXPERT for quantifying the amount of DNA and the purity was checked by measuring the 260/280 nm ratio. DNA samples were also subjected to agarose gel electrophoresis and, after passing through DNA quality check (Supplementary Table S6 and Supplementary Fig. S4), were proceeded for Library protocol.

### Whole-exome sequencing and bioinformatic analysis

The whole-exome sequencing library was prepared using Agilent-Sure Select XT Reagent Kit, Illumina (ILM) platforms. The sequencing was carried out in Illumina HiSeq X10 to generate 2X-150 bp sequence reads at an average 100 × sequencing depth to cover the maximum genomic variations. The quality check of WES-generated reads has been presented in Supplementary Tables S7, S8 and Supplementary Fig. S5. The alignment statistics and coverage analysis and average depth have been presented in Supplementary Tables S9, S10 and Supplementary Fig. S6, respectively. The bioinformatics analyses were achieved for variant calling and filtering with the major databases as GnomAD, ExAC, 1000G and ClinVar. The bioinformatic pipelines, alignment variant calling (Supplementary Fig. S7) and variant annotation (Supplementary Fig. S8) were processed and analyzed using the BROAD Institute’s GATK-Toolkit^34^. The variant calling was performed using the complete human reference genome (GRCh37/hg19). ClinVar database was used to check the previously reported mutations and associated phenotypes. Exclusion of intronic, synonymous, inframe insertions/deletions (InDels) and mutations in untranslated regions whereas the missense, nonsense variations and frameshift InDels located in exons or splice sites were prioritized. The remaining variants were then verified in dbSNP and NCBI databases. The WES methodology and detailed report have been presented in Supplementary material S1. The runs of homozygosity (ROH) was estimated using AutoMap^35^ and HomozygosityMapper^36^.

### In silico evaluation for the pathogenicity of candidate variant

The altered amino acid was checked for its evolutionary conservation across different species, including the primates and mammals, using the genome browser of the University of California at Santa Cruz (UCSC)^37^. Multiple in silico prediction algorithms, including CADD, FATHMM, MetaLR, MetaSVM, MutationTaster, PolyPhen-2 and SIFT were used to predict the possible impact of the detected variants.

## Supplementary Information

Supplementary Information.

## Data Availability

Web resources: ClinVar, https://www.ncbi.nlm.nih.gov/clinvar/. GenIO, http://bioinformatics.ibioba-mpsp-conicet.gov.ar/GenIO/index.php/. Mutation taster, http://www.mutationtaster.org/. NCBI dbSNP, https://www.ncbi.nlm.nih.gov/snp/. neXtProt, www.nextprot.org. OMIM, https://www.omim.org/. PolyPhen-2 http://genetics.bwh.harvard.edu/pph2/. SIFT, http://sift.jcvi.org/. STRING, https://stringdb.org/. SuSPect, http://www.sbg.bio.ic.ac.uk/~suspect/. The Human Gene Mutation Database, http://www.hgmd.cf.ac.uk/ac/index.php/. UniProtKB, https://www.uniprot.org/. Genome Aggregation Database (gnomAD) v2.1.1, https://gnomad.broadinstitute.org/. University of California Santa Cruz Genomics Institute Genome Browser, https://genome.ucsc.edu/.
