# Supplementary material for: Whole-exome sequencing reveals a novel homozygous mutation in the COQ8B gene associated with nephrotic syndrome
Source: Sci Rep. 2021 Jun 25;11:13337. doi: 10.1038/s41598-021-92023-3 (PMC8233304; doi:10.1038/s41598-021-92023-3)
Supplement: Supplementary file 1 — Supplementary Information. [file 41598_2021_92023_MOESM1_ESM.doc]

**Supplementary material S1**

**Whole-exome sequencing reveals a novel homozygous mutation in the *COQ8B* gene associated with nephrotic syndrome**

Mohd Fareed^1,2*^, Vikas Makkar^3^, Ravi Angral^4^, Mohammad Afzal^5^, Gurdarshan Singh^1,2^

^1^PK-PD Formulation & Toxicology Division, CSIR Indian Institute of Integrative Medicine, Canal Road, Jammu, 180001, India

^2^Academy of Scientific & Innovative Research (AcSIR), Ghaziabad, Uttar Pradesh, 201002, India

^3^Department of Nephrology, Dayanand Medical College and Hospital, Ludhiana, Punjab, 141001, India

^4^Visiting Consultant Renal Transplant, Dayanand Medical College and Hospital, Ludhiana, Punjab, 141001, India

^5^Human Genetics & Toxicology Laboratory, Section of Genetics, Department of Zoology, Aligarh Muslim University, Aligarh, Uttar Pradesh, 202002, India

*Corresponding author (M. Fareed) Tel.: +91-9622025886

Email address: [mohdfareedk@gmail.com](mailto:mohdfareedk@gmail.com); [m.fareed@iiim.res.in](mailto:m.fareed@iiim.res.in)

**Variant statistics of whole-exome sequencing data**

**Table S1. Overall pass-ontarget variant distribution.**

| **Samples** | **Total** | **Total** | **Total** | **Total** | **Total** | **Ts/Tv** |
| --- | --- | --- | --- | --- | --- | --- |
|  | **Variants** | **SNPs** | **InDels** | **Heterozygous** | **Homozygous** |  |
|  |  |  |  |  |  |  |
| MC9_2 | 73,706 | 64,697 | 9,009 | 43,366 | 30,340 | 2.30 |
|  |  |  |  |  |  |  |
| MC7_2 | 72,335 | 64,067 | 8,268 | 43,309 | 29,026 | 2.34 |
|  |  |  |  |  |  |  |
| MC8_1 | 72,669 | 64,564 | 8,105 | 44,059 | 28,610 | 2.34 |
|  |  |  |  |  |  |  |
| MC5_1 | 72,979 | 64,577 | 8,402 | 44,029 | 28,950 | 2.33 |
|  |  |  |  |  |  |  |
| MC4_1 | 73,139 | 64,778 | 8,361 | 44,600 | 28,539 | 2.32 |
|  |  |  |  |  |  |  |
| MC3_1 | 72,243 | 64,082 | 8,161 | 42,937 | 29,306 | 2.33 |
|  |  |  |  |  |  |  |
| MC2_1 | 72,731 | 64,562 | 8,169 | 44,881 | 27,850 | 2.36 |
|  |  |  |  |  |  |  |

**Table S2. Distribution of pass-ontarget and non-synonymous annotated variants.**

| **Sample** | **Missense** | **Nonsense** | **Startloss** | **Stoploss** | **Frameshift-indel** | **Inframe-indel** |
| --- | --- | --- | --- | --- | --- | --- |
|  |  |  |  |  |  |  |
| MC9_2 | 10,865 | 137 | 53 | 61 | 354 | 275 |
|  |  |  |  |  |  |  |
| MC7_2 | 10,892 | 150 | 52 | 61 | 358 | 255 |
|  |  |  |  |  |  |  |
| MC8_1 | 10,968 | 150 | 56 | 60 | 371 | 264 |
|  |  |  |  |  |  |  |

| MC5_1 | 10,939 |  | 147 | |  | 58 |  | 57 |  | 354 |  | 271 |  |  |
| --- | --- | --- | --- | --- | --- | --- | --- | --- | --- | --- | --- | --- | --- | --- |
|  |  |  |  |  |  |  |  |  |  |  |  |  |  |  |
| MC4_1 | 10,870 |  | 153 | |  | 52 |  | 60 |  | 353 |  | 263 |  |  |
|  |  |  |  |  |  |  |  |  |  |  |  |  |  |  |
| MC3_1 | 10,753 |  | 149 | |  | 52 |  | 57 |  | 354 |  | 262 |  |  |
|  |  |  |  |  |  |  |  |  |  |  |  |  |  |  |
| MC2_1 | 10,956 |  | 150 | |  | 57 |  | 64 |  | 359 |  | 273 |  |  |
|  |  |  | |  |  |  |  |  |  |  |  |  |  |  |

| **Table S3. Position-wise distribution of pass-ontarget annotated variants.** | | | | | | | | | | | | |  |  |
| --- | --- | --- | --- | --- | --- | --- | --- | --- | --- | --- | --- | --- | --- | --- |
|  |  | |  |  |  | |  | |  | |  | |  | |
| **Sample** | **Intergenic** | |  | **Exonic** | **Intronic** | | **Exonic-** | | **Exonic-** | | **Intronic-3-** | | **Intronic-5-** | |
|  |  |  |  |  |  |  | **5UTR** | | **3UTR** | | **Splice site** | | **Splice site** | |
|  |  |  |  |  |  | |  | |  | |  |  |  |  |
| MC9_2 | 1,245 |  |  | 55,024 | 17,436 | | 4,210 | | 24,477 | | 1,354 |  | 1,004 |  |
|  |  |  |  |  |  | |  | |  | |  |  |  |  |
| MC7_2 | 1,161 |  |  | 54,221 | 16,952 | | 4,032 | | 24,047 | | 1,296 |  | 943 |  |
|  |  |  |  |  |  | |  | |  | |  |  |  |  |
| MC8_1 | 1,165 |  |  | 54,613 | 16,890 | | 4,201 | | 23,866 | | 1,283 |  | 985 |  |
|  |  |  |  |  |  | |  | |  | |  |  |  |  |
| MC5_1 | 1,170 |  |  | 54,739 | 17,069 | | 4,127 | | 24,035 | | 1,315 |  | 974 |  |
|  |  |  |  |  |  | |  | |  | |  |  |  |  |
| MC4_1 | 1,212 |  |  | 54,891 | 17,035 | | 4,150 | | 24,414 | | 1,286 |  | 957 |  |
|  |  |  |  |  |  | |  | |  | |  |  |  |  |
| MC3_1 | 1,180 |  |  | 54,159 | 16,903 | | 3,982 | | 24,024 | | 1,300 |  | 971 |  |
|  |  |  |  |  |  | |  | |  | |  |  |  |  |
| MC2_1 | 1,181 |  |  | 54,473 | 17,076 | | 4,053 | | 24,007 | | 1,285 |  | 980 |  |
|  |  |  |  |  |  |  |  |  |  |  |  |  |  |  |

A total of 438 specific genes associated with the nephrotic disorder used for filtration.

**Table S4. Overall nephrotic related genes variant distribution.**

| **Samples** | **Total Variants** | **Total** | **Total** | **Total** | **Total** |
| --- | --- | --- | --- | --- | --- |
|  |  | **SNPs** | **InDels** | **Heterozygous** | **Homozygous** |
|  |  |  |  |  |  |
|  |  |  |  |  |  |
| MC2_1 | 244 | 223 | 21 | 144 | 100 |
| MC3_1 | 237 | 211 | 26 | 129 | 108 |
| MC4_1 | 236 | 216 | 20 | 126 | 110 |
| MC5_1 | 240 | 220 | 20 | 121 | 119 |
| MC7_2 | 245 | 221 | 24 | 127 | 118 |
| MC8_1 | 234 | 217 | 17 | 149 | 85 |
| MC9_2 | 241 | 220 | 21 | 139 | 102 |

**Table S5.** **Summary of likely pathogenic variants extracted from nephrotic gene panel.**

| **GENE_NAME** | **VAR_CLASS** | **CDNA_CHG** | **AA_CHG** | **SIFT (score)** | **1000G_Overall_af** | **ZYGOSITY** |  |
| --- | --- | --- | --- | --- | --- | --- | --- |
| *ACE* | 3UTR | c.*163C>A |  |  |  | Heterozygous | |
| *ACSF3* | MISSENSE | c.119C>T | p.Ser40Leu | T (0.2) |  | Heterozygous | |
| *AVPR2* | 5UTR | c.-153_-152insC |  |  |  | Homozygous | |
| *BBS9* | 3UTR | c.*775_*782dup |  |  | 0.00638978 | Heterozygous | |
| *BMP4* | 3UTR | c.*148del |  |  |  | Heterozygous | |
| *CD2AP* | 3UTR | c.*1368T>G |  |  |  | Heterozygous | |
| *CEP41* | 3UTR | c.*505G>T |  |  |  | Heterozygous | |
| *CLCNKA* | MISSENSE | c.23G>A | p.Arg8His | T_lc (0.06) | 0.0121805 | Heterozygous | |
| *CLCNKB* | MISSENSE | c.1877G>A | p.Cys626Tyr | D (0) | 0.0167732 | Heterozygous | |
| *CLCNKB* | 3UTR | c.*170G>T |  |  |  | Heterozygous | |
| *CLCNKB* | 3UTR | c.*194A>G |  |  |  | Heterozygous | |
| *CLCNKB* | 3UTR | c.*331C>G |  |  |  | Heterozygous | |
| *CLDN19* | 3UTR | c.*1018G>T |  |  | 0.0197684 | Heterozygous | |
| *COL4A3* | 3UTR | c.*1070dup |  |  |  | Heterozygous | |
| *COL4A4* | 3UTR | c.*2120dup |  |  |  | Heterozygous | |
| *COL4A5* | MISSENSE | c.3029A>G | p.Asn1010Ser | T (0.1) | 0.00741722 | Heterozygous | |
| *COQ7* | 3UTR | c.*143dup |  |  |  | Heterozygous | |
| *COQ8B* | MISSENSE | c.1000G>A | p.Asp334Asn |  |  | Homozygous | |
| ***COQ8B*^#$^** | MISSENSE | c.748G>A | p.Asp250Asn | D (0) |  | Homozygous | |
| *COQ9* | MISSENSE | c.362T>C | p.Ile121Thr | D (0) | 0.00139776 | Heterozygous | |
| *CREBBP* | 3UTR | c.*1478del |  |  |  | Homozygous | |
| *CTNS* | 3UTR | c.*687dup |  |  |  | Heterozygous | |
| *CTNS* | 3UTR | c.*688T>G |  |  |  | Heterozygous | |
| *EMP2* | 3UTR | c.*1149_*1156del |  |  |  | Heterozygous | |
| *EMP2* | 3UTR | c.*1153_*1156del |  |  |  | Heterozygous | |
| *EMP2* | 3UTR | c.*702del |  |  |  | Homozygous | |
| *ENPP1* | 3UTR | c.*3792del |  |  |  | Heterozygous | |
| *EYA1* | 3UTR | c.*1310G>A |  |  | 0.0101837 | Heterozygous | |
| *FGFR1* | 3UTR | c.*1026T>C |  |  | 0.000199681 | Heterozygous | |
| *FREM2* | MISSENSE | c.4319C>A | p.Thr1440Lys | D (0) | 0.00519169 | Heterozygous | |
| *FREM2* | MISSENSE | c.4990G>A | p.Ala1664Thr | D (0.02) | 0.00159744 | Heterozygous | |
| *GATA3* | 3UTR | c.*265dup |  |  |  | Heterozygous | |
| ***GLA*^$^** | MISSENSE | c.473C>T | p.Thr158Ile | D (0) |  | Heterozygous | |
| *KIAA0586* | MISSENSE | c.4243A>T | p.Met1415Leu | T (0.25) | 0.00159744 | Heterozygous | |
| *KIF14* | MISSENSE | c.2797A>G | p.Met933Val | T (0.43) |  | Heterozygous | |
| *KLHL3* | 3UTR | c.*3204A>T |  |  |  | Heterozygous | |
| *KLHL3* | 3UTR | c.*1451A>G |  |  | 0.0127796 | Heterozygous | |
| *KLHL3* | 3UTR | c.*319_*320del |  |  |  | Homozygous | |
| *LAMB2* | MISSENSE | c.2974A>G | p.Ile992Val | D (0.02) | 0.000399361 | Heterozygous | |
| *MAFB* | 3UTR | c.*377G>C |  |  |  | Heterozygous | |
| *MAGI2* | 3UTR | c.*93C>A |  |  |  | Heterozygous | |
| *MMACHC* | 3UTR | c.*213_*217dup |  |  |  | Homozygous | |
| *NOTCH2* | MISSENSE | c.137A>G | p.Asn46Ser | T (0.09) |  | Heterozygous | |
| *NOTCH2* | MISSENSE | c.112G>A | p.Glu38Lys | T (0.05) |  | Heterozygous | |
| *NOTCH2* | MISSENSE | c.57C>G | p.Cys19Trp | T (0.18) |  | Heterozygous | |
| *NOTCH2* | 5UTR | c.-26_-18del |  |  |  | Heterozygous | |
| *NOTCH2* | 5UTR | c.-134G>C |  |  |  | Heterozygous | |
| *NOTCH2* | 5UTR | c.-143G>T |  |  |  | Heterozygous | |
| *NOTCH2* | 5UTR | c.-148G>A |  |  |  | Heterozygous | |
| *NR3C2* | 3UTR | c.*478del |  |  |  | Heterozygous | |
| ***NUP107*^$^** | MISSENSE | c.1781G>A | p.Cys594Tyr | D (0.01) |  | Heterozygous | |
| *PEX2* | 3UTR | c.*1601T>A |  |  | 0.0163738 | Heterozygous | |
| *PEX7* | 5UTR | c.-95T>C |  |  |  | Heterozygous | |
| *PODXL* | MISSENSE | c.856A>G | p.Ser286Gly | T (0.29) | 0.00119808 | Heterozygous | |
| *PRKCA* | 5UTR | c.-4dup |  |  |  | Heterozygous | |
| *PTPRO* | MISSENSE-SS-PRX | c.1777G>A | p.Val593Met | T (0.1) | 0.000998403 | Heterozygous | |
| *ROBO2* | MISSENSE | c.19C>A | p.Arg7Ser | T_lc (0.47) |  | Heterozygous | |
| *ROBO2* | MISSENSE | c.1552G>T | p.Val518Leu | T (0.11) | 0.000798722 | Heterozygous | |
| *ROBO2* | MISSENSE | c.1516G>T | p.Val506Leu |  | 0.000798722 | Heterozygous | |
| *SIX1* | 3UTR | c.*2861_*2862dup | |  |  | Homozygous | |
| *SIX1* | 3UTR | c.*264_*265del |  |  |  | Heterozygous | |
| *SIX1* | 3UTR | c.*265del |  |  |  | Heterozygous | |
| *SLC34A1* | MISSENSE | c.199G>A | p.Val67Ile | T (0.3) |  | Heterozygous | |
| *SLC34A1* | 3UTR | c.*129G>A |  |  |  | Heterozygous | |
| *SLC4A1* | 3UTR | c.*1721G>A |  |  | 0.0131789 | Heterozygous | |
| *SLC9A3* | MISSENSE | c.2395T>C | p.Cys799Arg | T_lc (1) |  | Homozygous | |
| *STK39* | 5UTR | c.-124_-110dup |  |  |  | Heterozygous | |
| *TAT* | 3UTR | c.*1693C>T |  |  |  | Heterozygous | |
| *TBX18* | MISSENSE | c.868G>A | p.Gly290Arg | D (0.03) | 0.0139776 | Heterozygous | |
| *THBD* | 3UTR | c.*1993T>C |  |  | 0.0081869 | Heterozygous | |
| *TMEM216* | 3UTR | c.*21A>G |  |  | 0.00199681 | Heterozygous | |
| *XPO5* | 3UTR | c.*1353A>G |  |  | 0.00239617 | Heterozygous | |

**^#^**Pathogenic candidate homozygous variant.

**^$^**Novel variants.

*T* Tolerated, *T_lc* Tolerated low confidence, *D* Deleterious


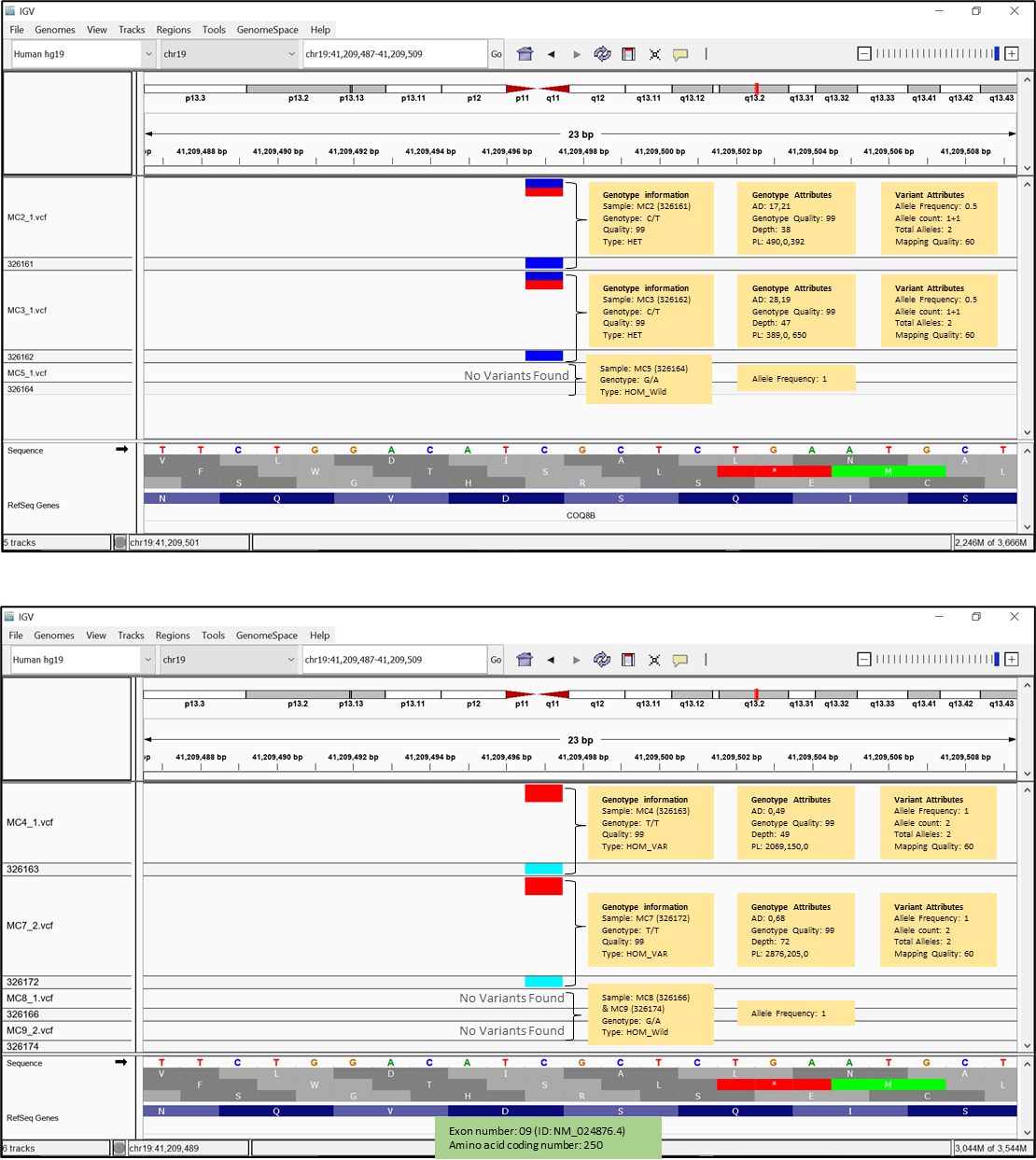


**Figure S1. Genotypic data.** Whole-exome data using VCF files in the Integrated Genomics Viewer (IGV) depicting the genotypic information of *COQ8B* candidate variant in all seven samples.


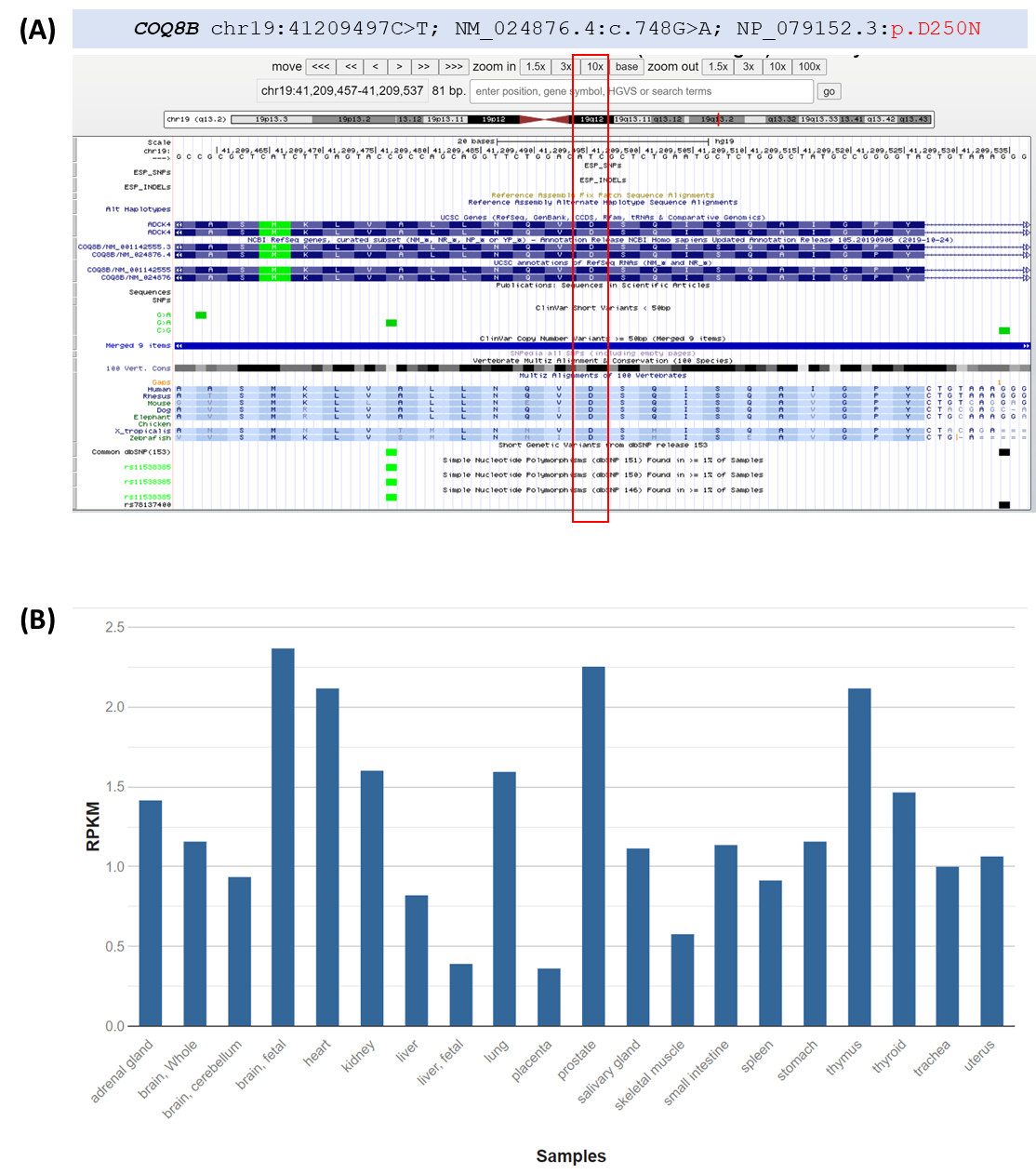


**Figure S2.** **COQ8B protein conservation and expression.** (A) Screenshot from UCSC Genome Browser showing the conserved region of *COQ8B (ADCK4)* gene via Multiz alignments of 100 vertebrates (B) *COQ8B* transcription profiling by high throughput sequencing of individual and a mixture of 20 human tissues RNA (Source: NCBI, Gene ID: 79934, BioProject: PRJNA280600, updated: 24-11-2020).

**Clinical observations**


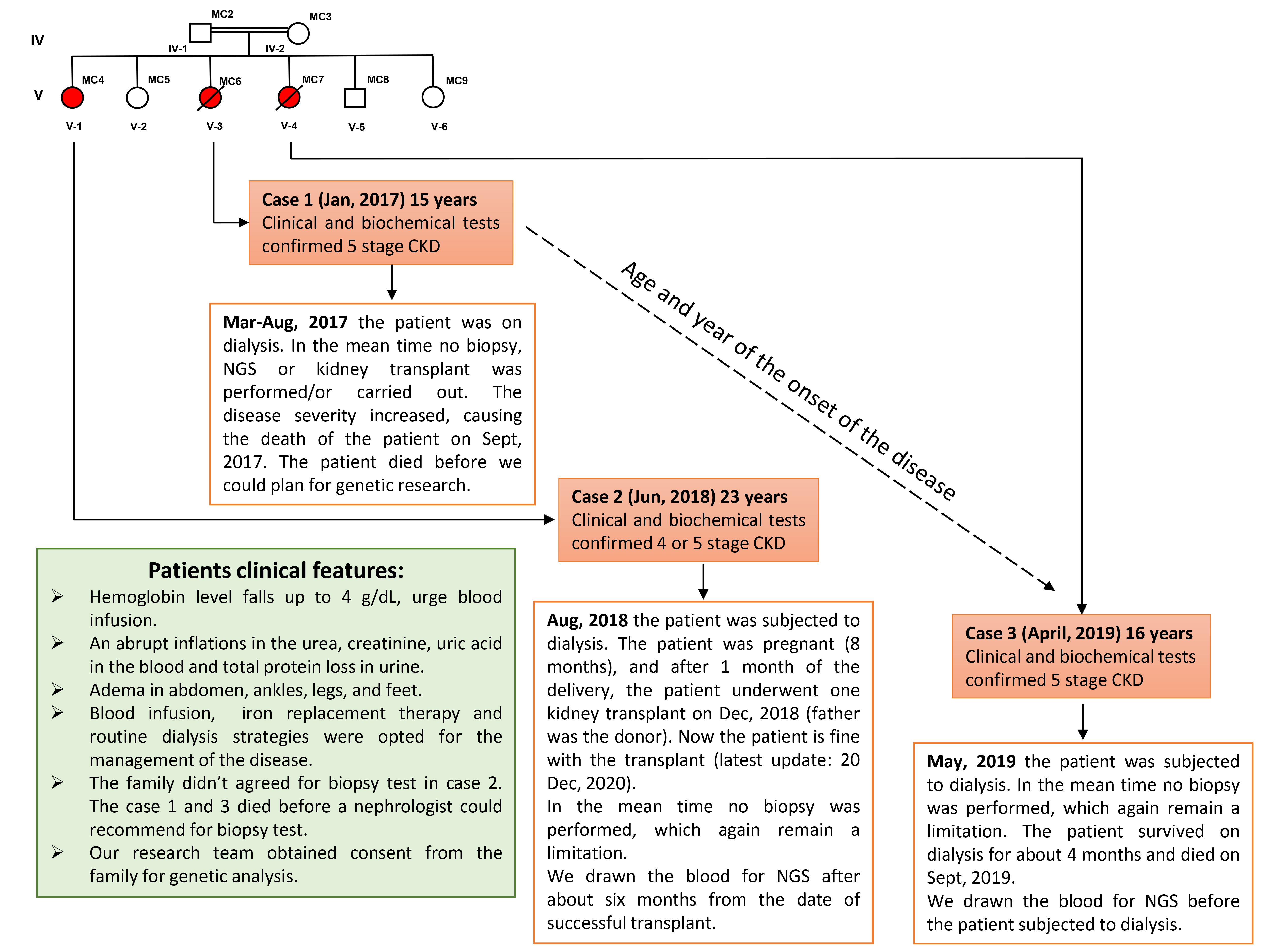


**Figure S3. Detailed history, clinical characteristics and disease manifestation of the patients.** The patients remained asymptomatic until they reached the most advanced stage of CKD. Two subjects (case-1 and case-3) died with ESRD, while case-2 was rescued with a kidney transplant. The clinical characteristics show resemblance with steroid nephrotic resistance syndrome (SRNS).

**Whole-exome sequencing**

**(a) Methodology**

**DNA Sample QC**

DNA samples were subjected to QIAXPERT and Qubit for quantifying the amount of DNA in the extracted sample and also the 260/280nm ratio was looked upon for the purity of the same. They were also subjected for Agarose Gel electrophoresis. All the 7 samples passed the QC criteria and were proceeded for library protocol.

**DNA Library Prep Protocol:**

Whole-Exome sequencing libraries were prepared using Agilent-Sure Select XT Reagent Kit, Illumina (ILM) platforms (Cat.No: G9641B). Biotinylated oligonucleotide capture probes (V5+UTR (Cat no. 5190-6214)), also called as baits, that was designed for the human exons was provided with the kit and used to enrich the region of interest (whole exome) by hybridization. The workflow involved shearing of DNA, repairing ends, adenylation of 3’ ends, followed by adapter ligation. At each step the products were purified using AMPure beads (Beckman Coulter, Cat.No:A63882). The adapter sequence was added onto the ends of DNA fragments to generate paired-end libraries. The resulting adaptor-ligated libraries were purified, qualified and hybridized with an exome-specific biotinylated capture library. After hybridization, the targeted molecules were captured on streptavidin beads. The resulting enriched DNA libraries were multiplexed by adding index tags by amplification, followed by purification. Indexed captured library DNAs were assessed to check the quality and quantity of the captured libraries.

**Sequencing Protocol:**

Prepared libraries were quantified using Qubit High Sensitivity reagent. The obtained libraries were diluted to final concentration of 2nm in 10 ul and was subjected for Cluster amplification. Once the cluster generation was completed, the flow cell was loaded on to the sequencer. The Sequencing was carried out in Hi Seq X sequencer to generate 2X150 bp sequence reads at an average 80-100X sequencing depth. A minimum of 75% of the sequenced bases was of Q30 value. Sequenced data was processed to generate FASTQ, BAM, VCF files further analysis.

**Table S6.** DNA Sample QC report.

|  |  |  |  |  |  |  |  |  |  |  |  |  |  |  |  |  |
| --- | --- | --- | --- | --- | --- | --- | --- | --- | --- | --- | --- | --- | --- | --- | --- | --- |
|  |  |  |  |  | **Qiaexpert** | | | **A260/280** | | |  | **Qubit ng/µl** |  |  | **Comments** |  |
|  | **Sample ID** |  | **Sample Name** |  |  | **(ng/µL)** |  |  | **RATIO** |  |  |  |  |  | **(PASS/FAIL)** |  |
|  |  |  |  |  |  |  |  |  |  |  |  |  |  |  |  |  |
|  |  |  |  |  |  |  |  |  |  |  |  |  |  |  |  |  |
| 326161 | |  | MC2_1 | | 72.9 | |  | 1.78 | |  | 47.2 | |  |  | PASS | |
|  |  |  |  |  |  |  |  |  |  |  |  |  |  |  |  |  |
| 326162 | |  | MC3_1 | | 50.9 | |  | 1.82 | |  | 36.6 | |  |  | PASS | |
|  | |  |  | |  | |  |  | |  |  | |  |  |  | |
| 326163 | |  | MC4_1 | | 97.3 | |  | 1.8 | |  | 71.8 | |  |  | PASS | |
|  | |  |  | |  | |  |  | |  |  | |  |  |  | |
| 326164 | |  | MC5_1 | | 142 | |  | 1.85 | |  | 108 | |  |  | PASS | |
|  | |  |  | |  | |  |  | |  |  | |  |  |  | |
| 326165 | |  | MC7_1 | | 28.2 | |  | 1.76 | |  | 21.6 | |  |  | PASS | |
|  | |  |  | |  | |  |  | |  |  | |  |  |  | |
| 326166 | |  | MC8_1 | | 49.8 | |  | 1.82 | |  | 36 | |  |  | PASS | |
|  | |  |  | |  | |  |  | |  |  | |  |  |  | |
| 326167 | |  | MC9_1 | | 16.3 | |  | 1.64 | |  | 11 | |  |  | PASS | |
|  | |  |  | |  | |  |  | |  |  | |  |  |  | |
| 326168 | |  | MC2_2 | | 52.9 | |  | 1.8 | |  | 37.2 | |  |  | PASS | |
|  | |  |  | |  | |  |  | |  |  | |  |  |  | |
| 326169 | |  | MC3_2 | | 65.7 | |  | 1.78 | |  | 47.8 | |  |  | PASS* | |
|  | |  |  | |  | |  |  | |  |  | |  |  |  | |
| 326170 | |  | MC4_2 | | 116.4 | |  | 1.73 | |  | 85.2 | |  |  | PASS | |
|  | |  |  | |  | |  |  | |  |  | |  |  |  | |
| 326171 | |  | MC5_2 | | 22.3 | |  | 1.61 | |  | 13.8 | |  |  | PASS | |
|  | |  |  | |  | |  |  | |  |  | |  |  |  | |
| 326172 | |  | MC7_2 | | 49.7 | |  | 1.78 | |  | 35.6 | |  |  | PASS | |
|  | |  |  | |  | |  |  | |  |  | |  |  |  | |
| 326173 | |  | MC8_2 | | 43.1 | |  | 1.79 | |  | 30.6 | |  |  | PASS | |
|  | |  |  | |  | |  |  | |  |  | |  |  |  | |
| 326174 | |  | MC9_2 | | 67.5 | |  | 1.8 | |  | 57.6 | |  |  | PASS | |
|  |  |  |  |  |  |  |  |  |  |  |  |  |  |  |  |  |

**PASS*- Degraded DNA**

**A260/280** **absorbance wavelength ratio used to assess the purity of DNA**

**Note: 5 ul used for QC check from the whole elution volume**

**Note: All samples taken here in duplicates for quality check.**

**
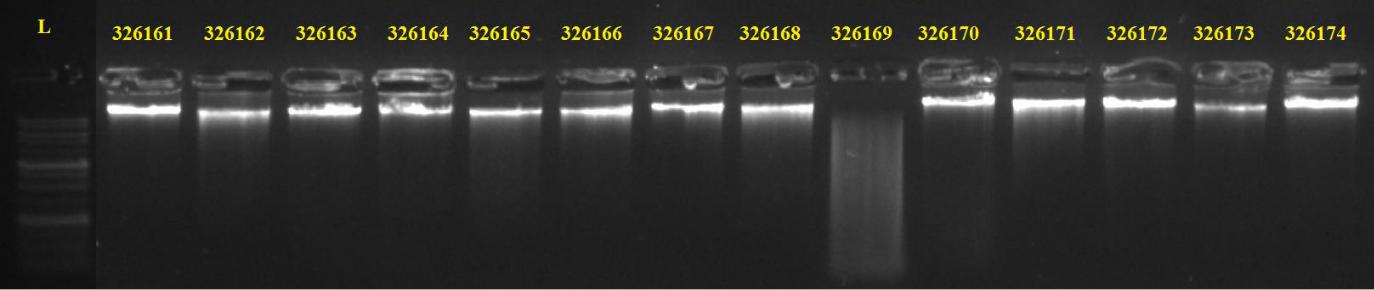
**

**Figure S4. DNA quantification using gel electrophoresis.**
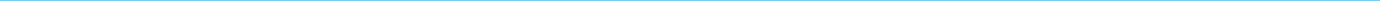
Samples loaded in duplicates.

**(b) Fastq File Quality Check Report**

**Table S7**. Raw reads summary.


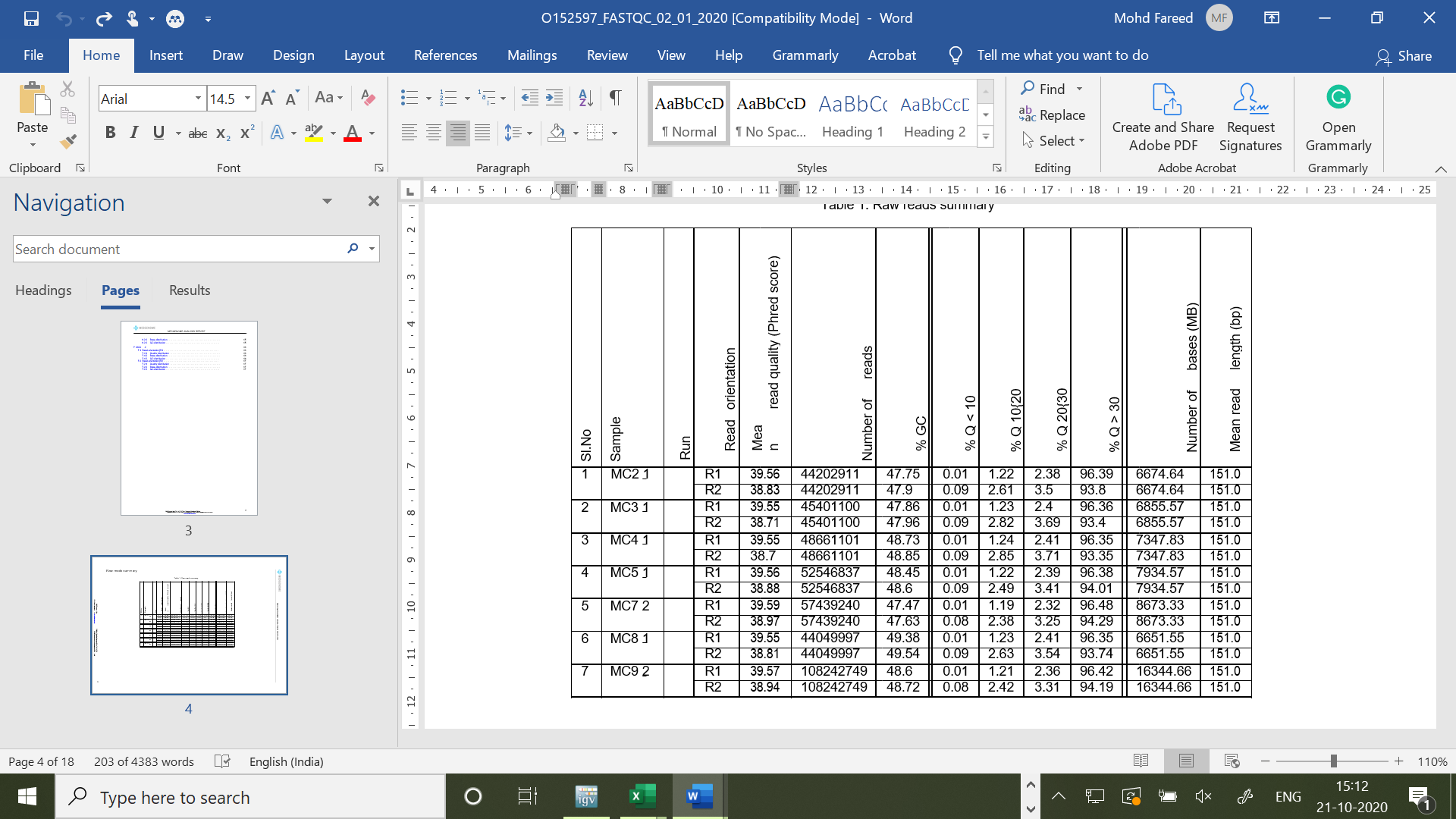


**(c) Whole-exome sequencing (WES) report**

The total data generated for the 7 samples was around 13.30 - 32.69 Gb. The quality score distribution indicates that at least 96.69% of data for each sample is above Q30. The overall alignment and the passed alignment percentage (alignment to hg19) in all the samples was around 99.98 and 97.09 percentage respectively. The analysis of the samples was performed after alignment using SSV5UTR panel (74,557,381 bp) which covers 23690 genes. The on-target percentage for the samples range from 84.14 - 85.91%. The average panel depth for each sample ranges from 102.55 to 247.33 X.

**SAMPLES**

| S.No | Sample |
| --- | --- |
|  |  |
| 1 | MC9_2 |
|  |  |
| 2 | MC7_2 |
|  |  |
| 3 | MC8_1 |
|  |  |
| 4 | MC5_1 |
|  |  |
| 5 | MC4_1 |
|  |  |
| 6 | MC3_1 |
|  |  |
| 7 | MC2_1 |
|  |  |

**1. Quality Check and Data Summary**

**Table S8.** **Raw data summary (overall quality metrics of the data)**

| **Sample** | **Total** | **Total Data** | **GC%** | **Avg Base** | **%Data >** |
| --- | --- | --- | --- | --- | --- |
|  | **Reads** | **(Gb)** |  | **Quality** | **Q30** |
|  |  |  |  |  |  |
| MC9_2 | 216,485,498 | 32.689 | 48 | 38.761 | 97.121 |
|  |  |  |  |  |  |
| MC7_2 | 114,878,480 | 17.347 | 47 | 38.780 | 97.182 |
|  |  |  |  |  |  |
| MC8_1 | 88,099,994 | 13.303 | 49 | 38.691 | 96.912 |
|  |  |  |  |  |  |
| MC5_1 | 105,093,674 | 15.869 | 48 | 38.726 | 97.064 |
|  |  |  |  |  |  |
| MC4_1 | 97,322,202 | 14.696 | 48 | 38.641 | 96.687 |
|  |  |  |  |  |  |
| MC3_1 | 90,802,200 | 13.711 | 47 | 38.642 | 96.742 |
|  |  |  |  |  |  |
| MC2_1 | 88,405,822 | 13.349 | 47 | 38.701 | 96.947 |
|  |  |  |  |  |  |


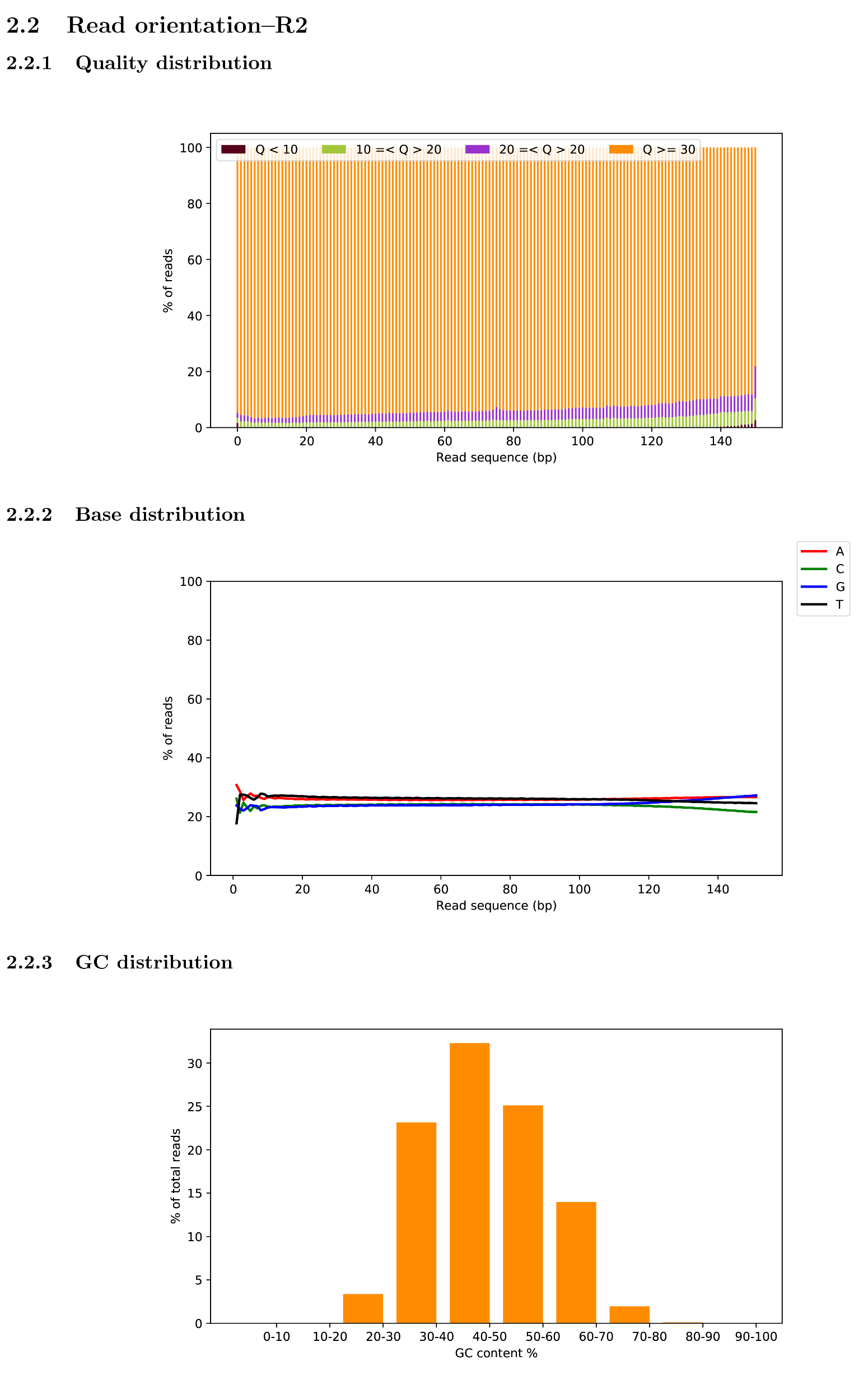

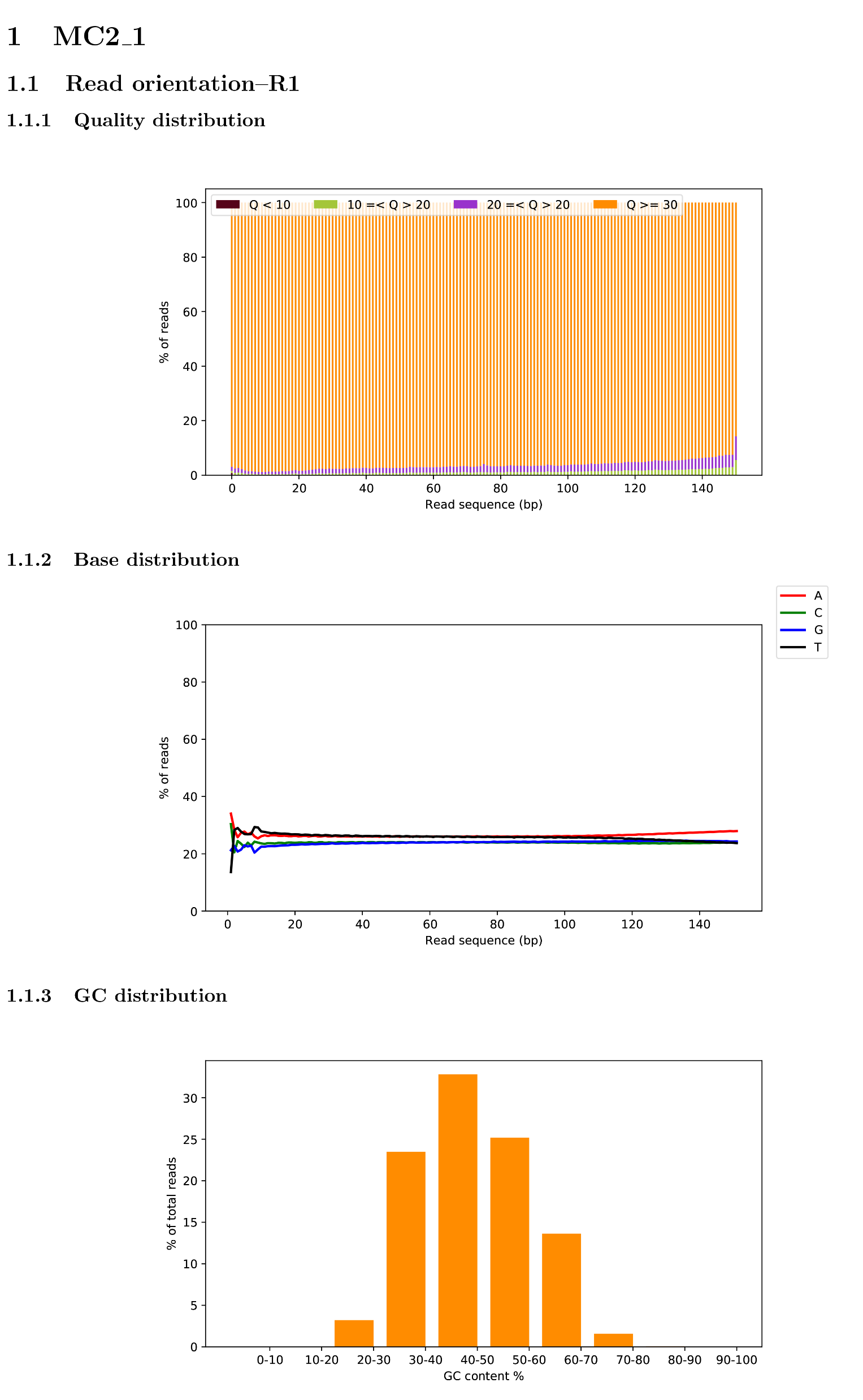

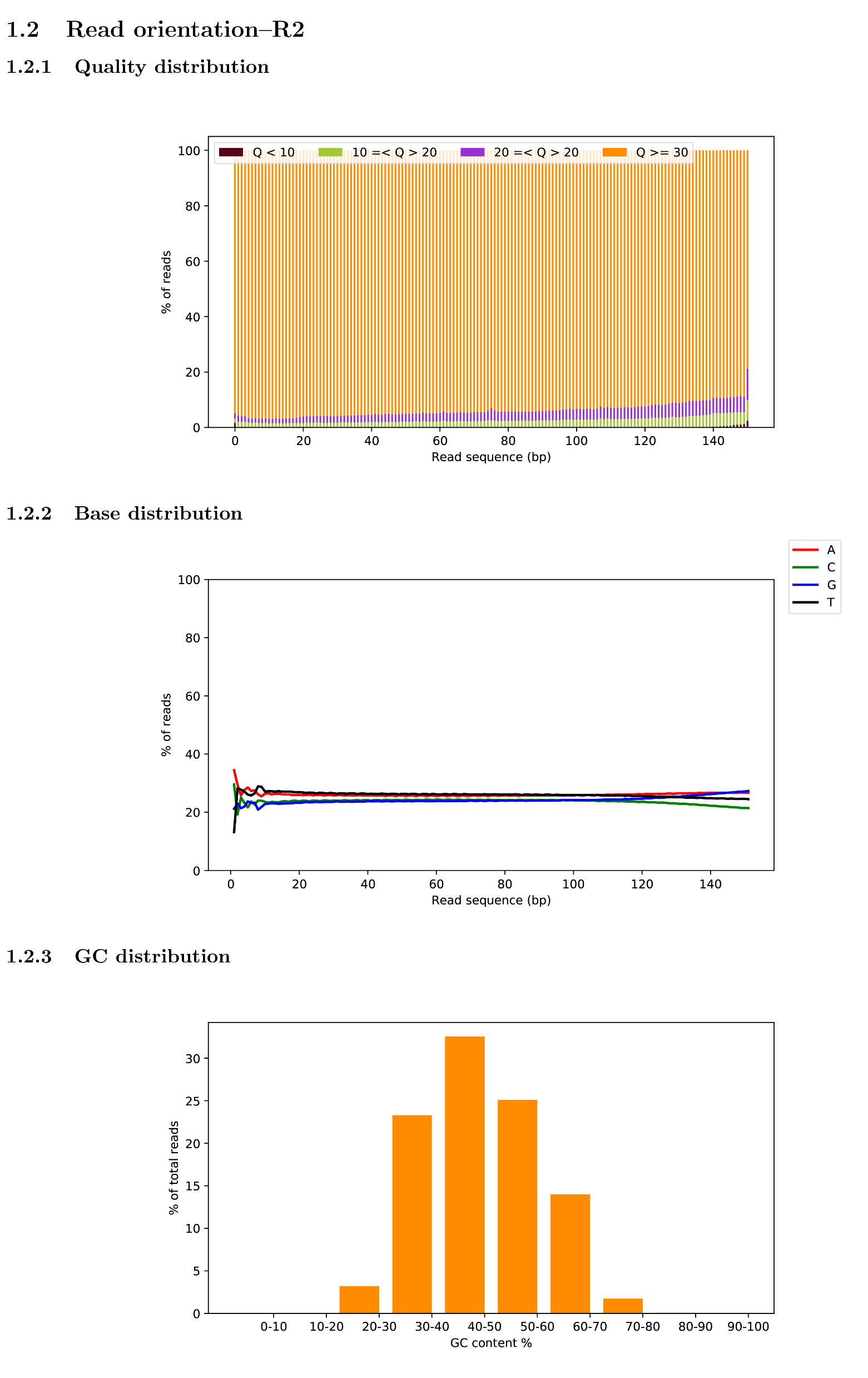

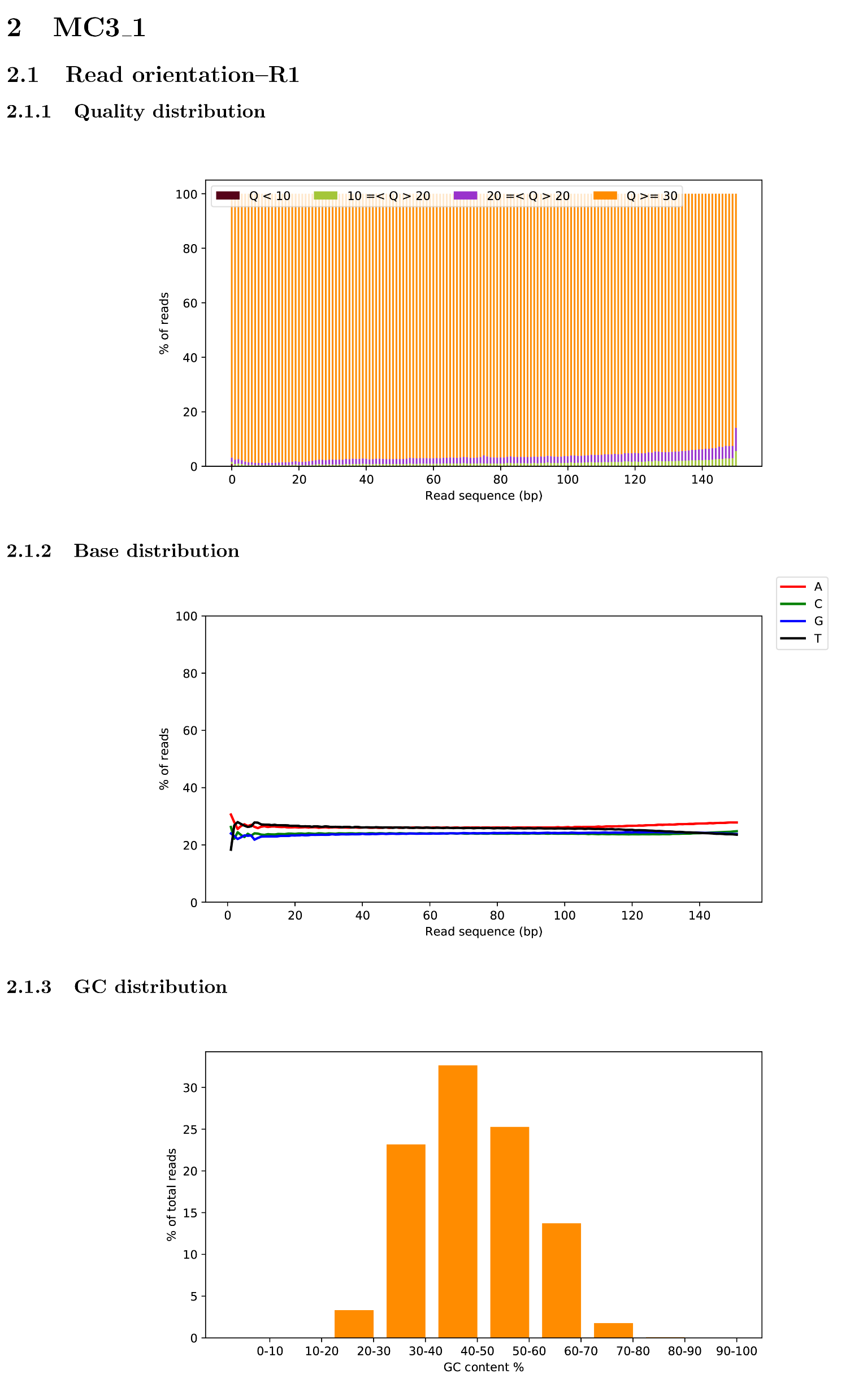


**Figure S5.** **Quality check of WES reads.** Data represent quality distribution, base distribution and GC distribution in all seven samples.


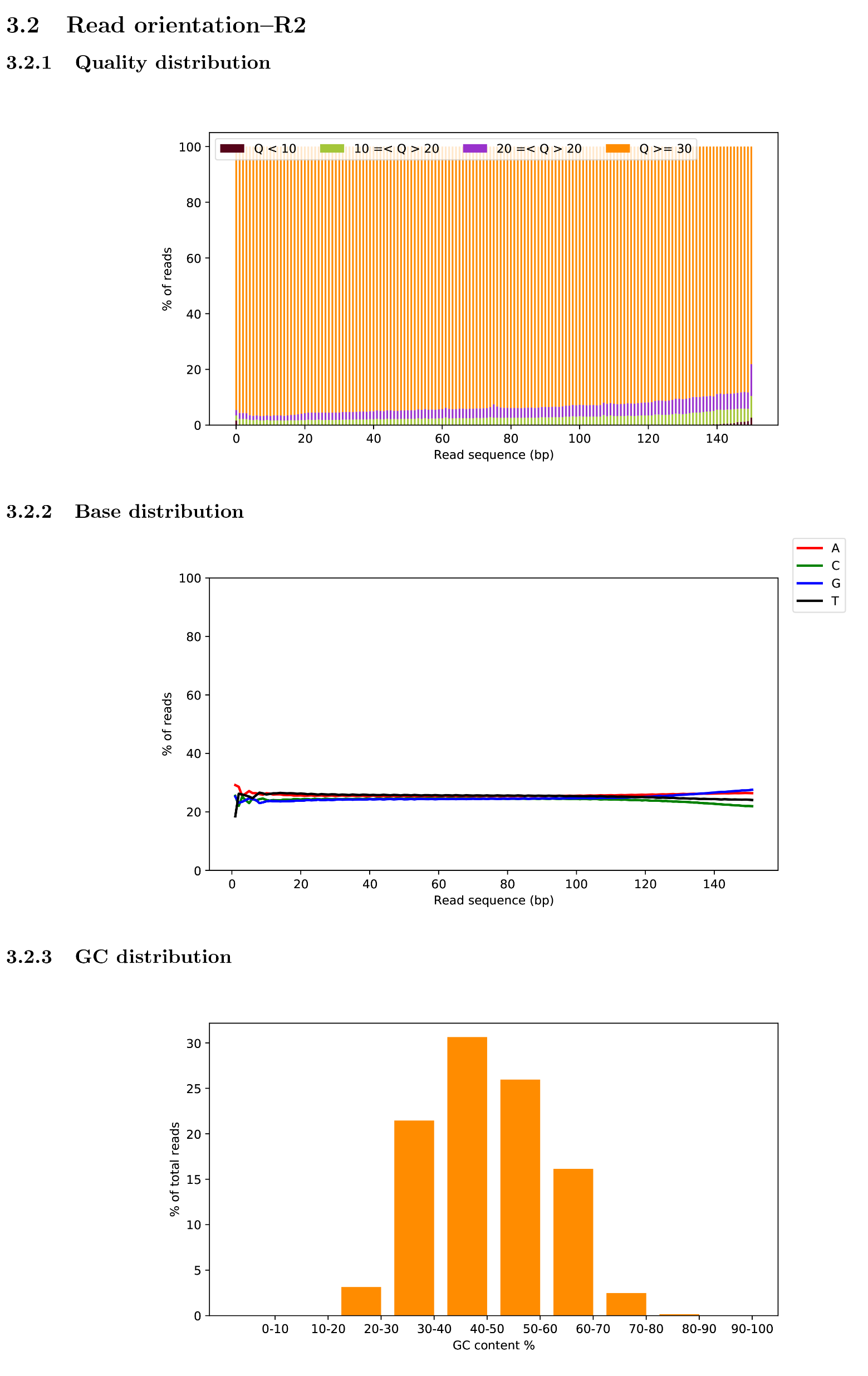

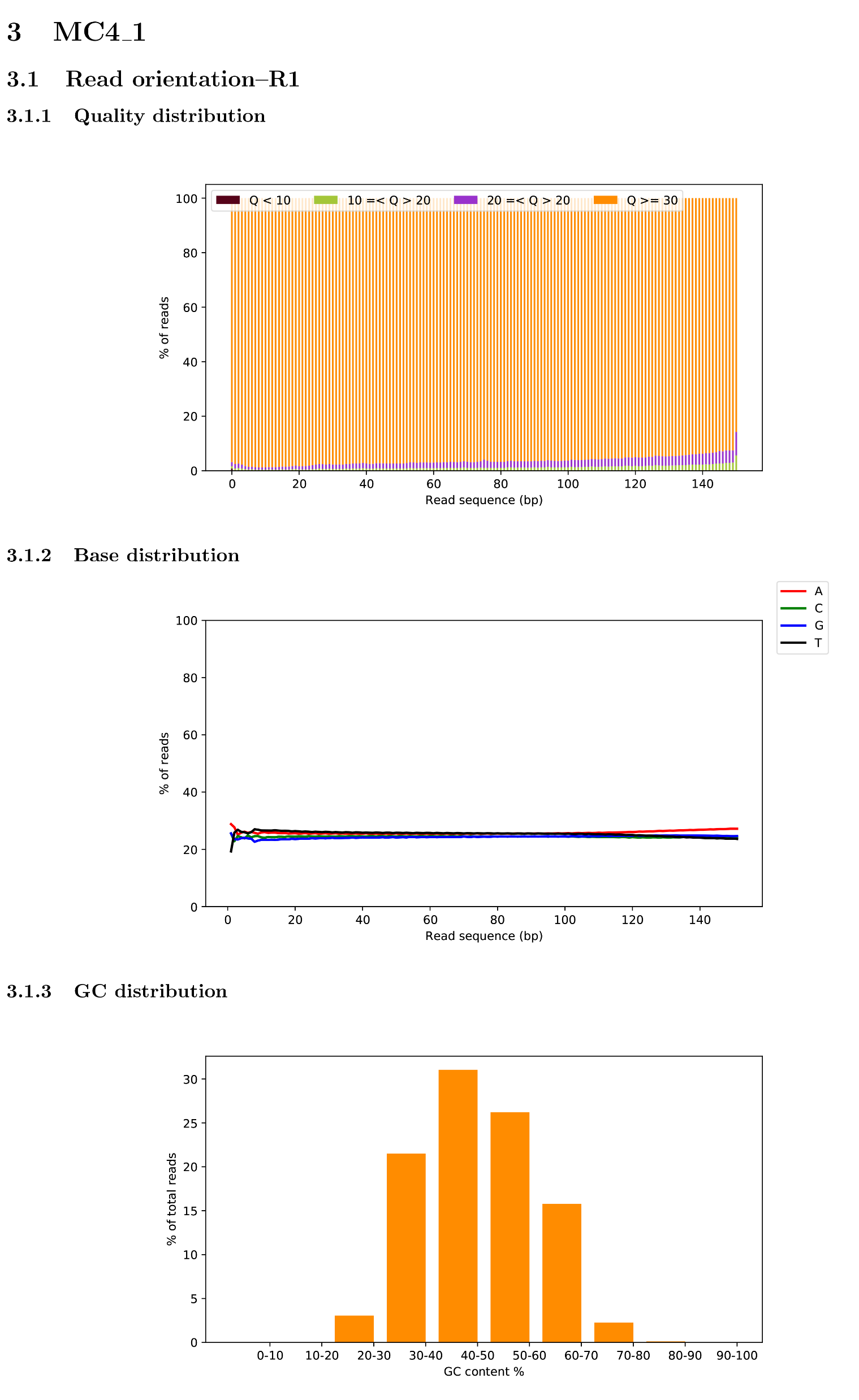

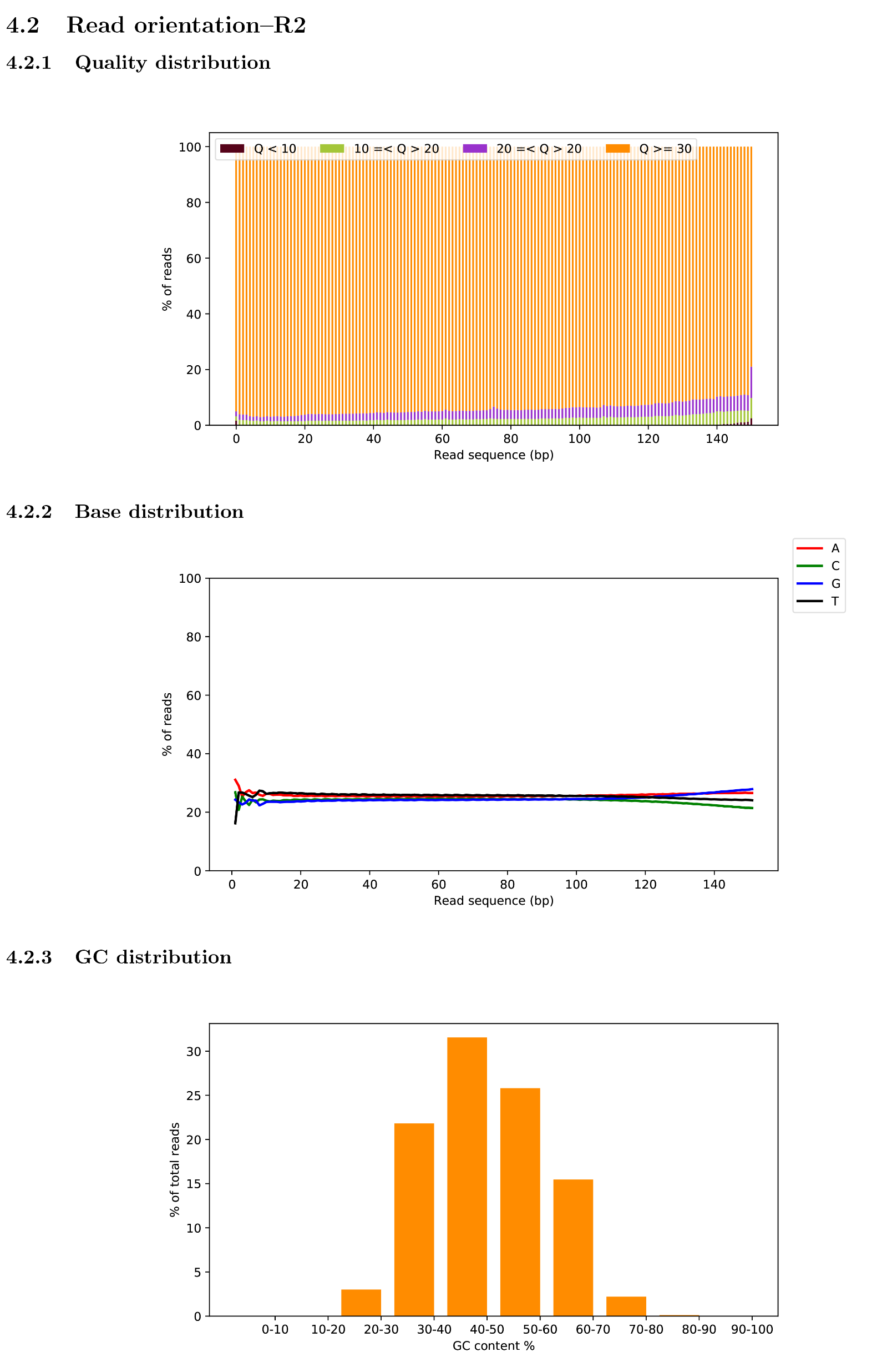

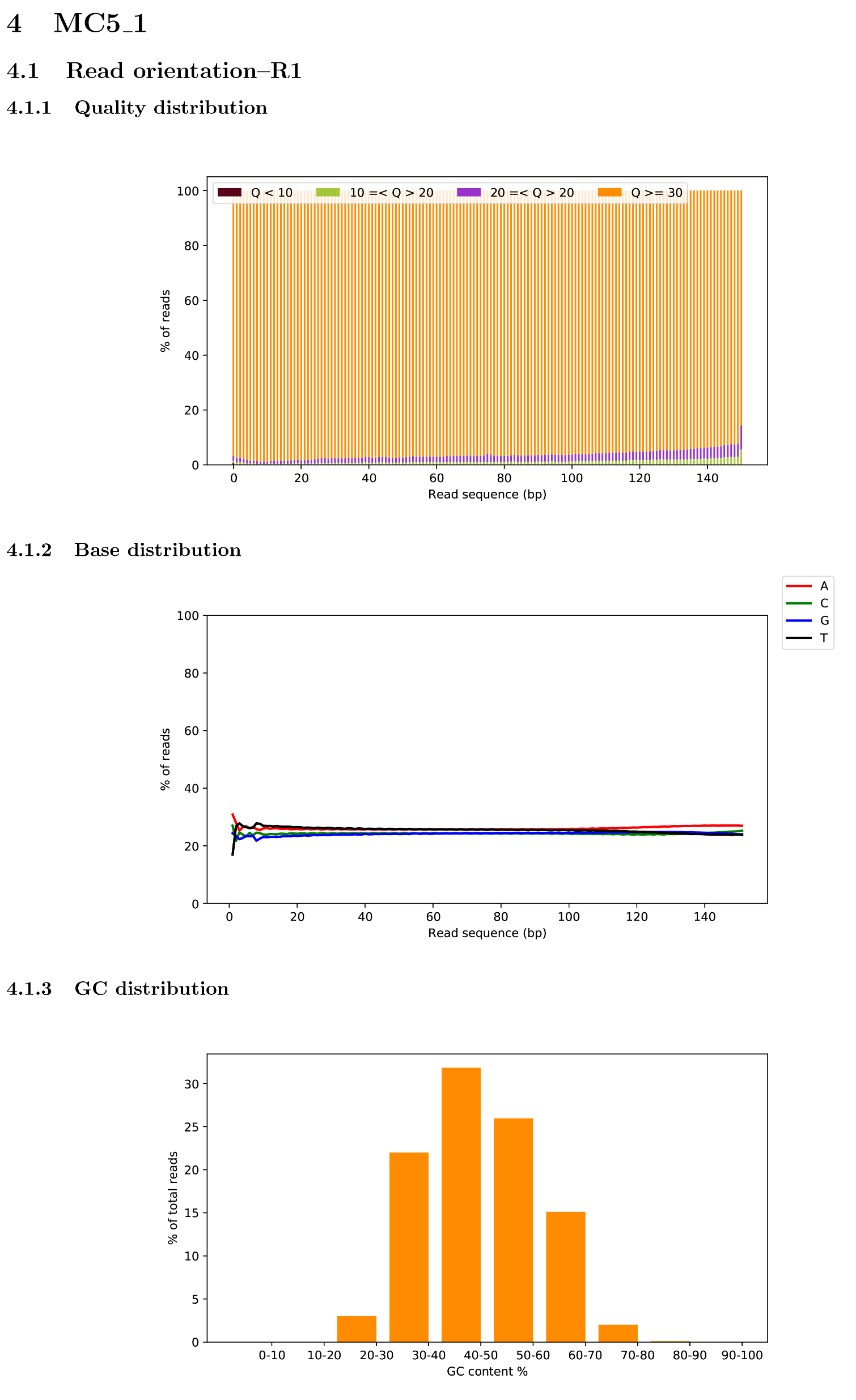


**Figure S5.** Continued.


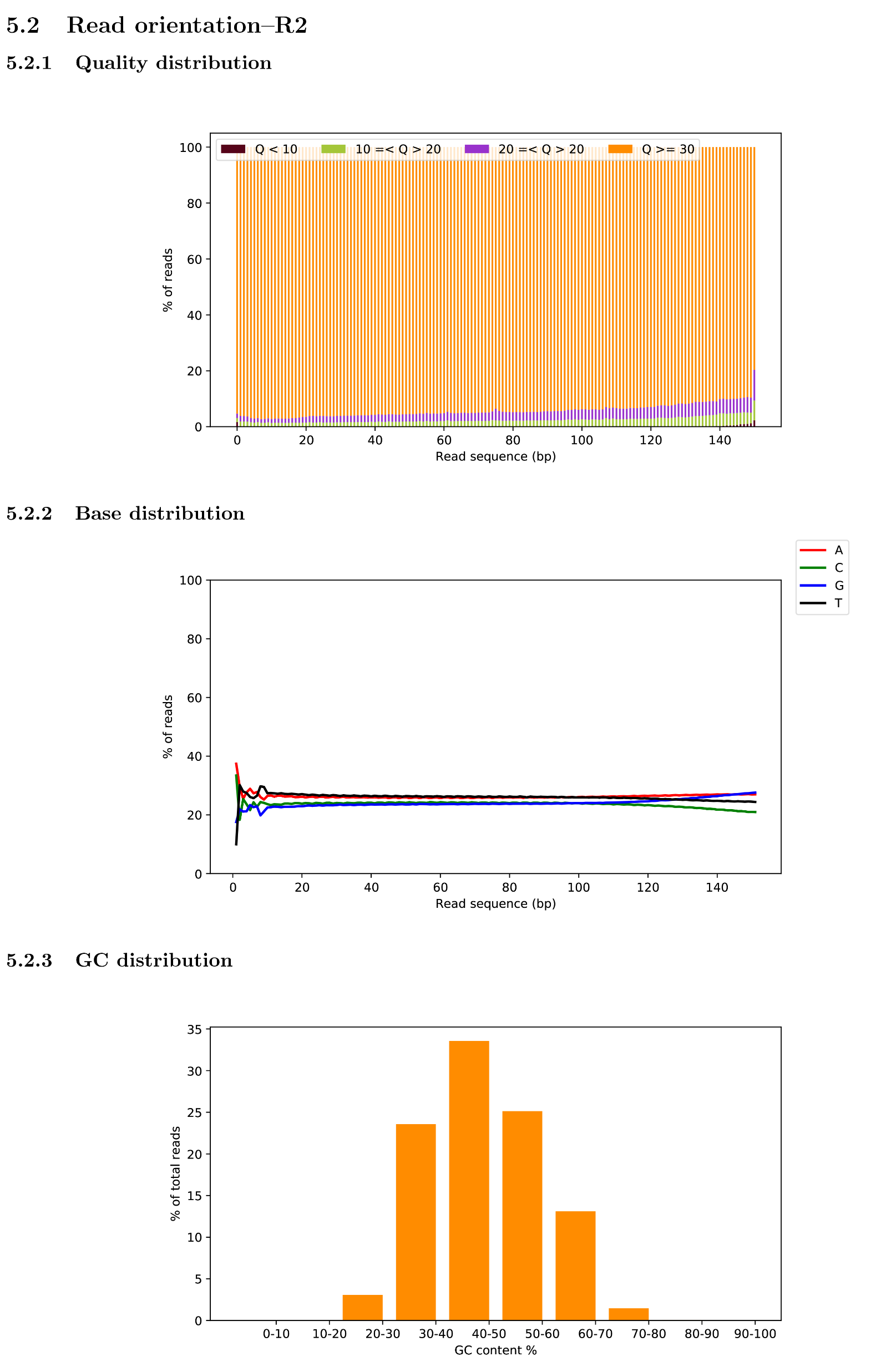

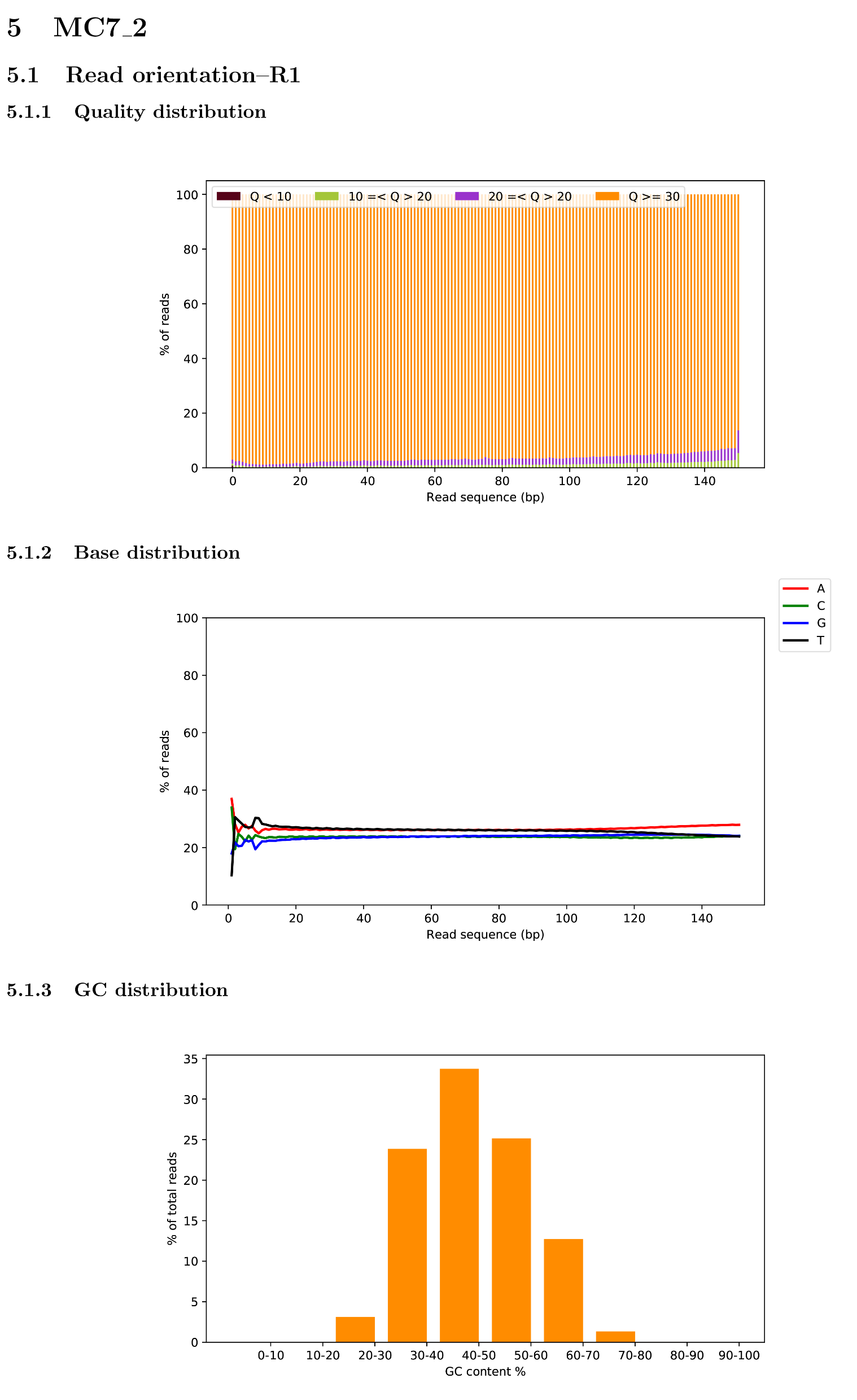

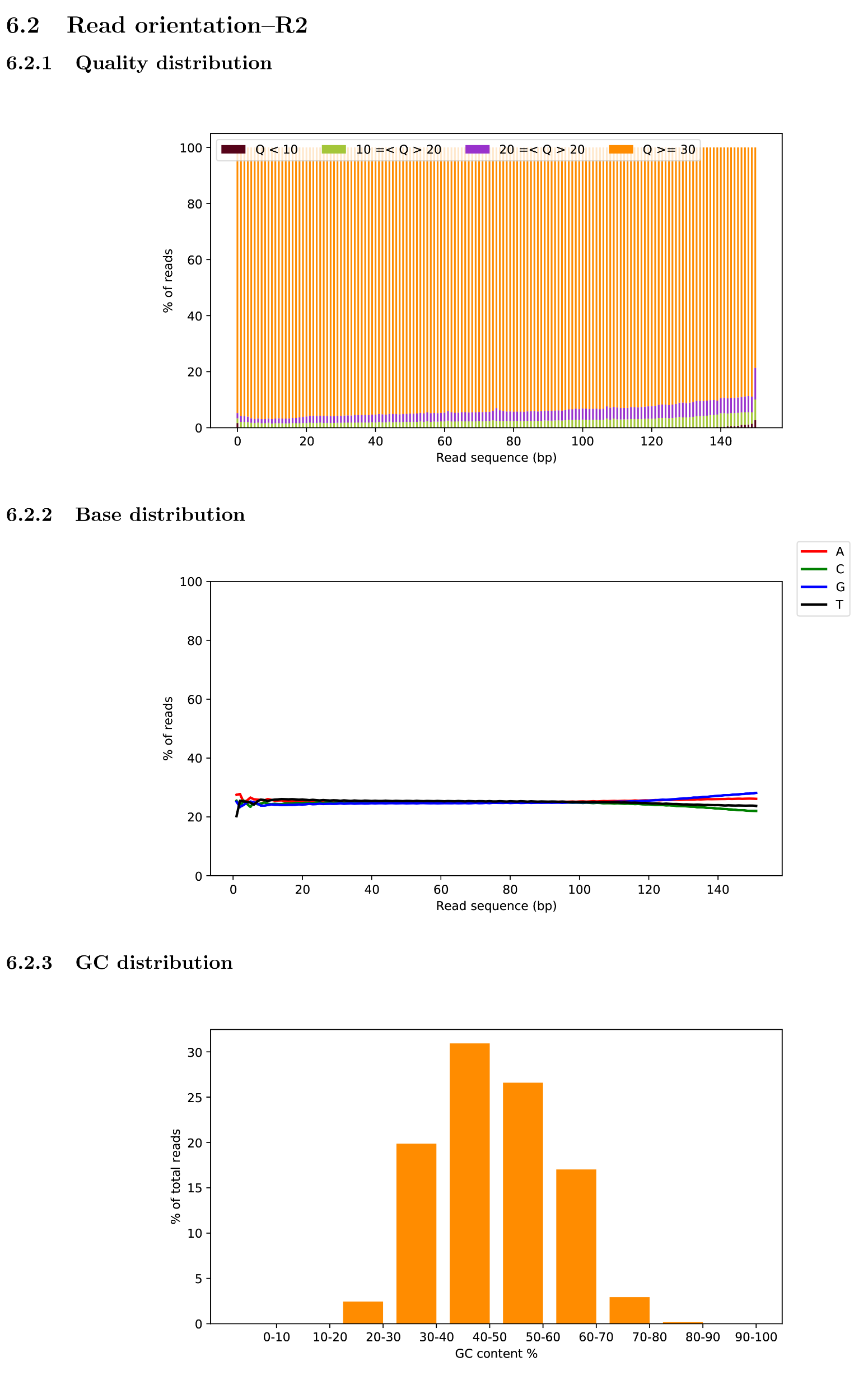

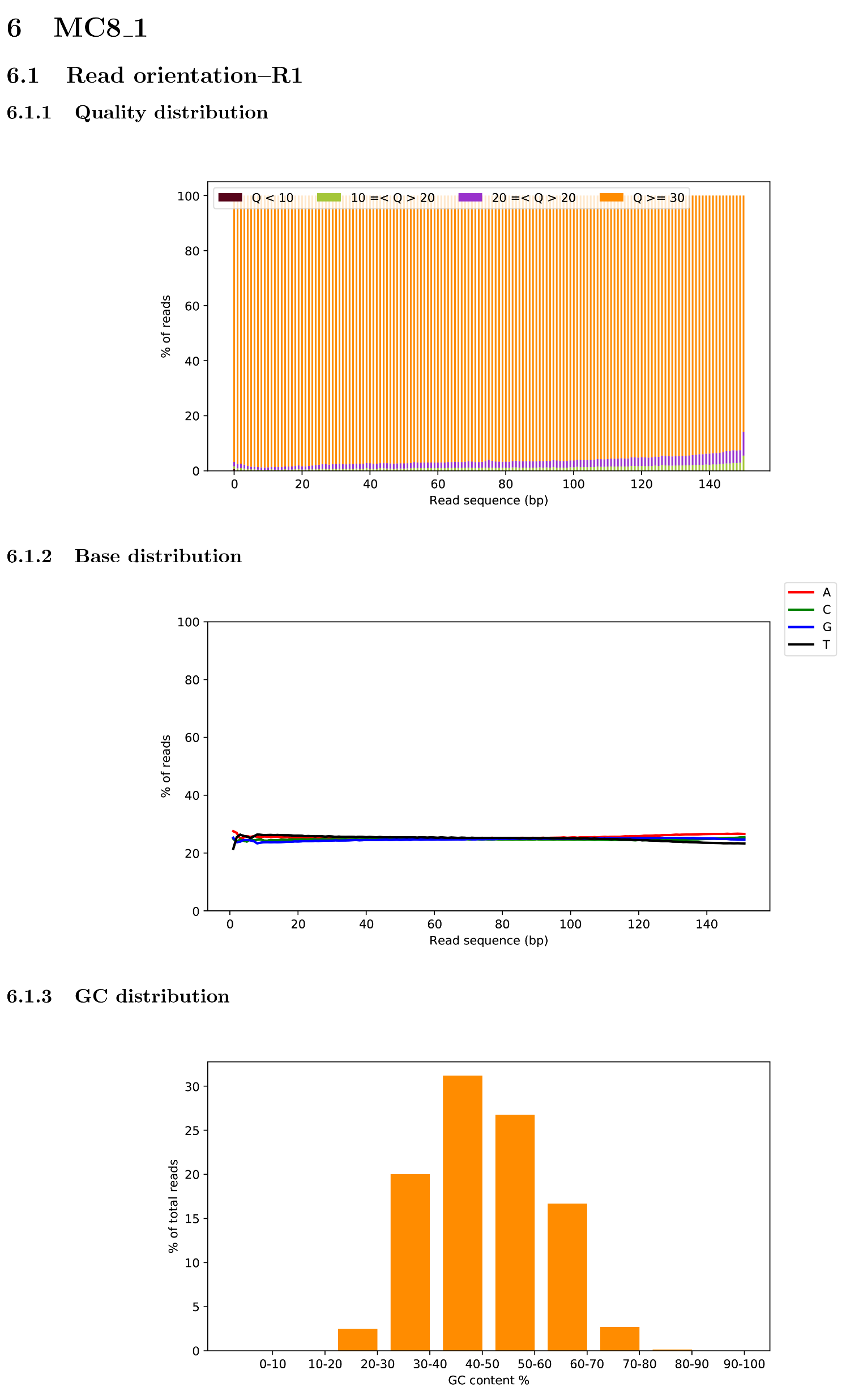


**Figure S5.** Continued.


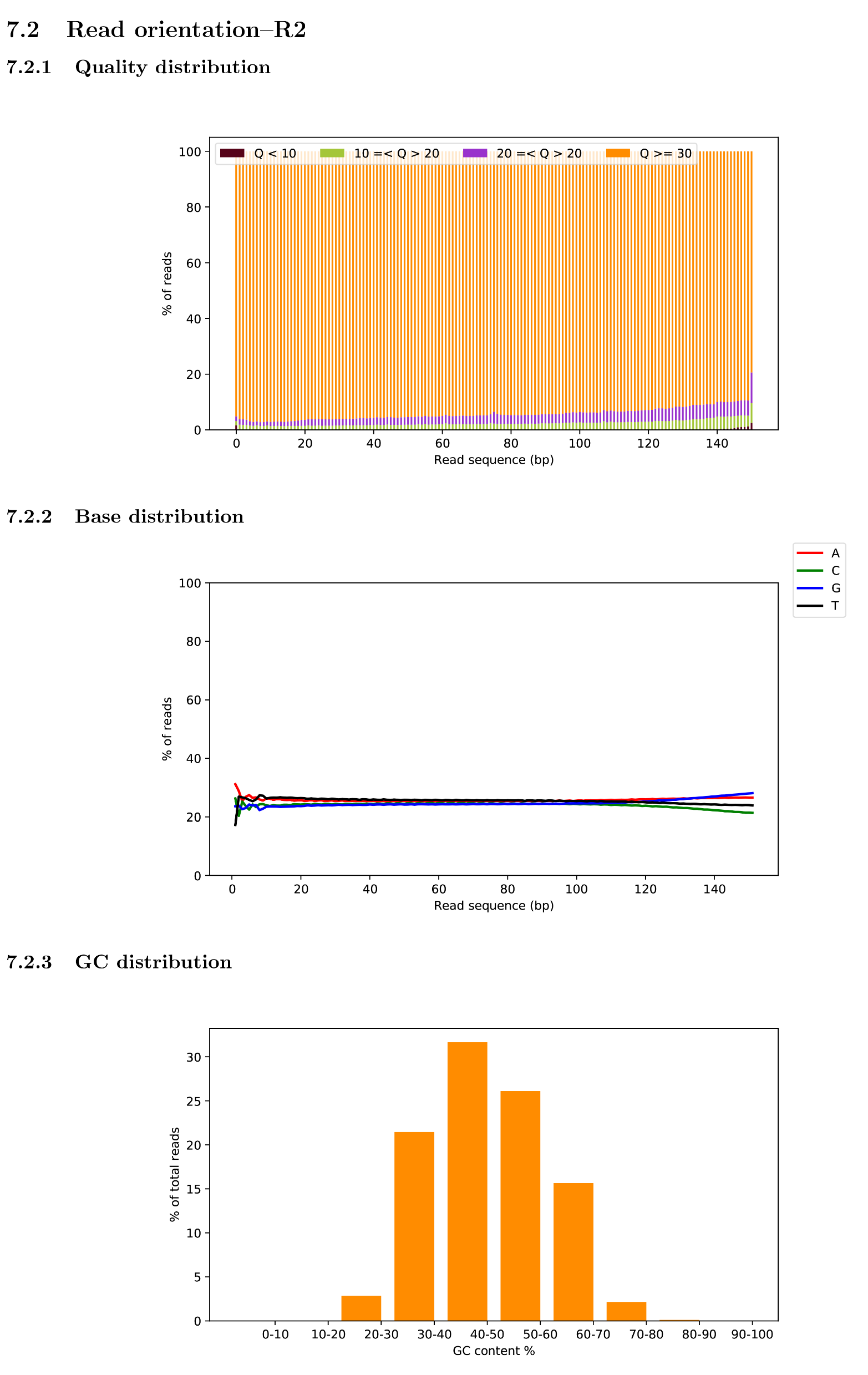

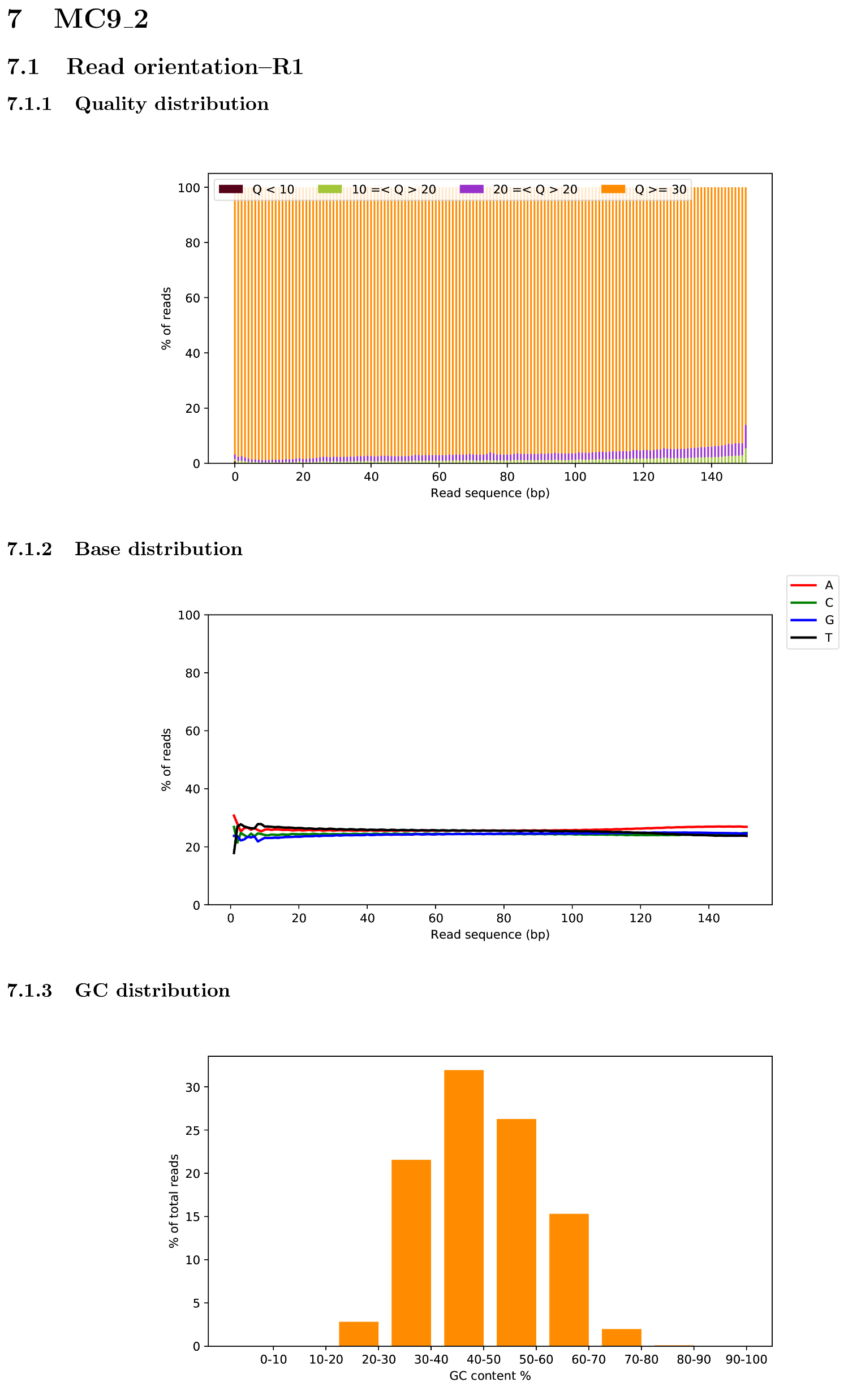


**Figure S5.** Continued.

**2. Alignment summary**

The overall alignment is around 99.98 percentage and the average passed alignment (percent of reads aligning to hg19) is around 97.09 percent for all the samples.

**Table S9: Alignment statistics**

| **Sample** | **Reads after adapter trimming** | **Total aligned reads** | **Total alignment %** | **Total passed alignment %** | **Total failed alignment %** | **Total crossmaped %** | **Total unaligned %** | **Duplicates %** |
| --- | --- | --- | --- | --- | --- | --- | --- | --- |
| MC9_2 | 196,367,274 | 196,352,792 | 99.993 | 97.279 | 2.721 | 0.305 | 0.009 | 10.402 |
| MC7_2 | 104,724,966 | 104,596,502 | 99.877 | 96.257 | 3.743 | 0.695 | 0.135 | 7.882 |
|  |  |  |  |  |  |  |  |  |
| MC8_1 | 79,994,696 | 79,988,518 | 99.992 | 97.441 | 2.559 | 0.209 | 0.010 | 8.189 |
|  |  |  |  |  |  |  |  |  |
| MC5_1 | 95,712,250 | 95,704,220 | 99.992 | 97.223 | 2.777 | 0.335 | 0.011 | 7.887 |
|  |  |  |  |  |  |  |  |  |
| MC4_1 | 89,413,592 | 89,406,658 | 99.992 | 97.259 | 2.741 | 0.266 | 0.010 | 7.588 |
|  |  |  |  |  |  |  |  |  |
| MC3_1 | 83,197,580 | 83,191,966 | 99.993 | 97.166 | 2.834 | 0.288 | 0.009 | 6.752 |
|  |  |  |  |  |  |  |  |  |
| MC2_1 | 81,318,854 | 81,314,008 | 99.994 | 97.024 | 2.976 | 0.446 | 0.008 | 7.093 |
|  |  |  |  |  |  |  |  |  |

**3. Coverage analysis**

The average coverage of the samples on the panel is around 99.24 percent. The on-target percentage for the samples is around 85.20 percent.

**Table S10. Panel Coverage**

| **Sample** | **Panel Coverage %** | **Panel Avg Depth** | **Read Ontarget %** |
| --- | --- | --- | --- |
|  |  |  |  |
| MC9_2 | 99.283 | 247.332 | 85.909 |
|  |  |  |  |
| MC7_2 | 99.161 | 133.704 | 85.060 |
|  |  |  |  |
| MC8_1 | 99.324 | 102.548 | 85.492 |
|  |  |  |  |
| MC5_1 | 99.216 | 123.378 | 85.628 |
|  |  |  |  |
| MC4_1 | 99.232 | 113.395 | 84.295 |
|  |  |  |  |
| MC3_1 | 99.204 | 106.205 | 84.137 |
|  |  |  |  |
| MC2_1 | 99.278 | 105.888 | 85.896 |
|  |  |  |  |


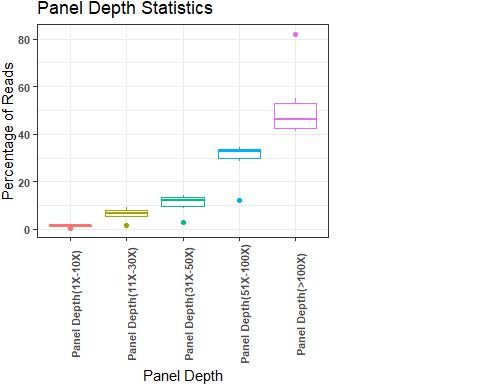


**Figure S6. Panel depth statistics.** Distribution of reads (in percentage) across ranges of depth.

**4. Analysis Overview**

The following bioinformatics steps were performed for the analysis of whole-exome data:


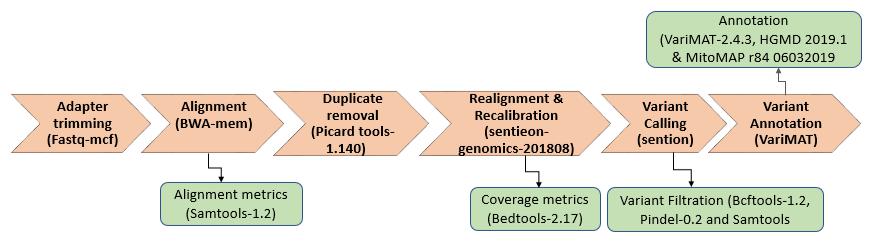


**Figure S7. Schematic representation of the bioinformatics analysis pipeline.** The workflow contains major components of the bioinformatics analysis pipeline and the tools used in each component.

**4.1. Adapter trimming**

The Adapter trimming phase involves processing of raw FASTQ files from the upstream analysis to remove adapters and generating QC metrics on raw and trimmed read data. The open-source fastq-mcf command line tool was used for detecting and removing the sequencing adapters, primers, poor quality nucleotides at the ends of reads.

**4.2. Alignment**

The Alignment phase involves aligning of trimmed FASTQ reads to a human genome reference sequence (hg19/GRCh37) and obtaining variants from the alignments. We follow GATK good-practice workflow steps for secondary analysis. Alignment of adapter trimmed FASTQ reads was performed using Sention’s version of BWA. The adapter trimmed FASTQ files were used as input for aligning with the human reference genome (hg19). The reference genome can be accessed at: [http://hgdownload.soe.ucsc.edu/goldenPath/hg19/bigZips/chromFa.tar.gz.](http://hgdownload.soe.ucsc.edu/goldenPath/hg19/bigZips/chromFa.tar.gz) Sorting and conversion of the alignment SAM file to binary compressed BAM file format was done using samtools.

**4.3. PCR Duplicates removal**

The BAM files from the alignment step were further sorted in coordinate order to perform PCR duplicates removal using Sentieon’s version of Picard tools and chromosome wise alignment metrics were generated.

**4.4. Indel realignment**

Sentieon’s version of Genome Analysis Toolkit (GATK - IndelRealigner) was used to perform local realignment in regions containing potential indels.

**4.5. Base quality score recalibration**

The raw Phred-scaled quality scores do not always accurately reflect the true base-calling error rate. To recalibrate the quality scores of all the reads in the BAM file, we use Sentieon’s version of GATK Toolkit - BaseRecalibrator. During this analysis step, the set of mapped reads at a locus is locally re-aligned to the primary human reference sequence.

**4.6. Variant calling**

The variant calling is a process of obtaining variants such as indels and SNPs by scanning through the aligned reads to reference sequence. The results were generated in VCF format using Sentieon’s GATK Haplotypecaller and UnfiedGenotyper. At a minimum, these files record information and annotations about the sequence variants identified, such as their type (e.g., SNP, Indel). Both haplotype caller and genotype caller variants files were merged into a single variant VCF file. Both the callers were used to avoid missing any variant. If the variant was common in both the callers, then the variant was picked from Haplotype Caller file. These variants were further processed for normalization using bcftools.

**4.7. Variant annotation**

The variant annotation pipeline (VariMAT - Variation and Mutation Annotation Toolkit) was used for variant annotation. It integrates multiple clinical-grade databases, variant class prediction and variants pathogenicity prediction tools for annotating the variants and mutation which rely on VEP. The VariMAT contains more than 70 entities for every transcript to annotate in-depth to understand their cause/effect on associated disease or phenotype. Some of the annotated information available in VariMAT were the population frequency, computational pathogenicity prediction, variant type and predicted impact of the variant on the protein (missense, loss of function, etc).


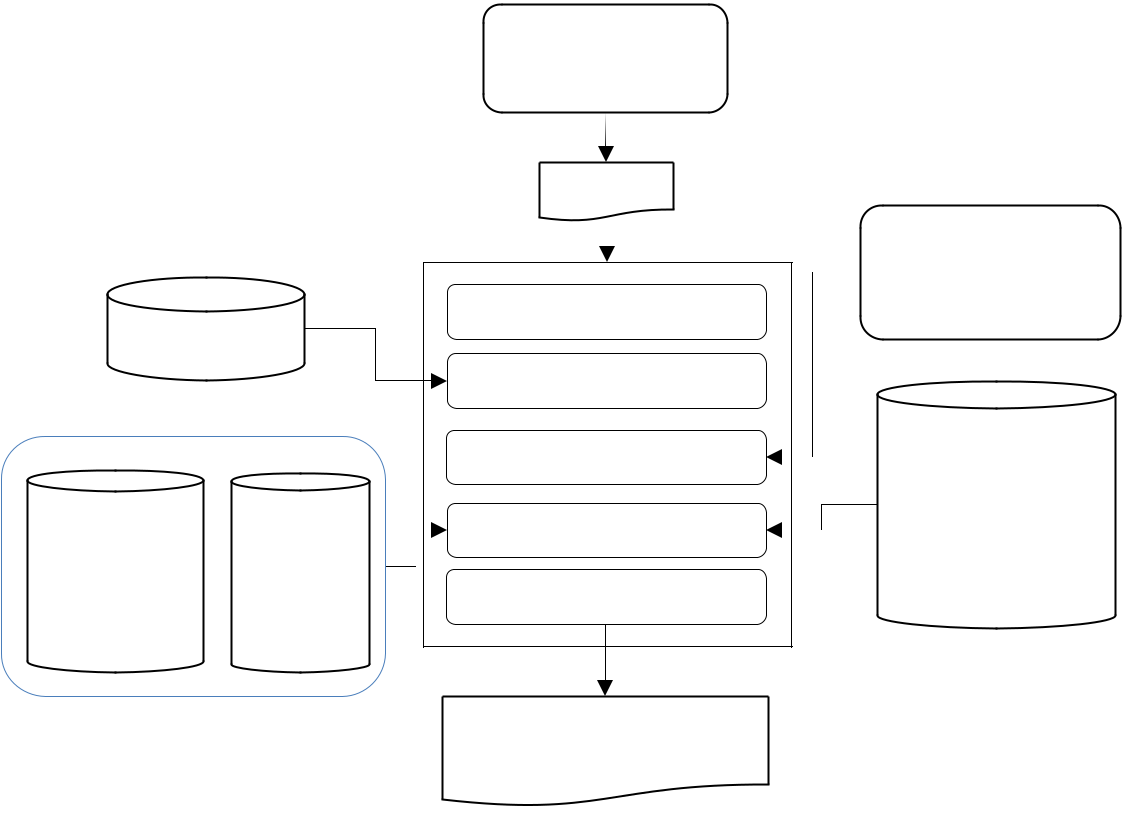


Secondary

analysis

VCF file

Panel

MedVarDb 1000G

TWINSUK dbSNP

|  | VEP |
| --- | --- |
| VCF processor | (EnsEMBL’s |
| OnTarget Variant |  |
| Gene Annotator | HGMD |
|  |
| Common Variant | OMIM |
|  |
| Disease Annotation | ClinVar |
|  |

VariMAT file –

Annotated Variant file

**Figure S8. Variant annotation pipeline.** VariMAT pipeline is the in-house variant annotation component of MedGenome’s bioinformatics analysis system (https://diagnostics.medgenome.com/).

**Software and Database versions**

| **Software** |  | **Version** |
| --- | --- | --- |
| QC_PROGRAM |  | split-qc-1.0 |
| FASTQ-MCF |  | ea-utils-1.1.2-806 |
| BWA-MEM |  | bwa-0.7.8-r455 |
| SAMTools |  | samtools-1.2 |
| SamFilteration |  | v1.1 |
| BCFTools |  | bcftools-1.2 |
| BEDTools |  | bedtools-2.17 |
| Sentieon |  | sentieon-genomics-201808 |
| HGMD |  | 2019.1 |
| rtg-tools |  | v3.8.4 |
| Reference | Genome | hg19/Grch37 |
| Version |  |  |
| Metrics (Coverage) | | V5.3-9thMarch2018 |
| JAVA |  | jdk1.8.0_91 |
| Picard Tools |  | picard-tools-1.140 |
| R |  | v3.5.0 |
| VariMAT |  | 2.4.3 |
| PhyloP7way |  | v2 |
| SIFT |  | v5.2.2 |
| PROVEAN |  | v1.1 |
| PolyPhen_2 |  | v2.2.2 |
| Mutation Assessor | | v3 |
| PhastCons7way |  | v2 |
| VCFTOOLS |  | v0.1.14 |

**5. WES supplementary files available on request:**

- Raw data – raw Fastq files (.fastq.gz)
- Vcf file – sample-wise variant files(.vcf)
- Annotation files – annotated variants (.VariantAnnotation.txt) filtered based on variant location (only on-target selected), variant quality (only passed variants selected) and canonical transcripts (presence of canonical transcript).
- Alignment files – BAM file with alignment of sample to reference genome (.bam)
- Filtered Annotation files - annotated variants filtered based on gene list provided by client.
